# Unlocking Antimicrobial Peptides: In Silico Proteolysis and Artificial Intelligence-Driven Discovery from Cnidarian Omics

**DOI:** 10.3390/molecules30030550

**Published:** 2025-01-25

**Authors:** Ricardo Alexandre Barroso, Guillermin Agüero-Chapin, Rita Sousa, Yovani Marrero-Ponce, Agostinho Antunes

**Affiliations:** 1 Interdisciplinary Centre of Marine and Environmental Research (CIIMAR/CIMAR), University of Porto, Terminal de Cruzeiros do Porto de Leixões, Av. General Norton de Matos s/n, 4450-208 Porto, Portugal; barrosoalex98@gmail.com (R.A.B.); gchapin@ciimar.up.pt (G.A.-C.); rmsds2013@gmail.com (R.S.); 2 Department of Biology, Faculty of Sciences of University of Porto (FCUP), Rua do Campo Alegre s/n, 4169-007 Porto, Portugal; 3 Facultad de Ingeniería, Universidad Panamericana, Augusto Rodin No. 498, Insurgentes Mixcoac, Benito Juárez, Ciudad de Mexico 03920, Mexico; ymarrero77@yahoo.es; 4 Grupo de Medicina Molecular y Traslacional (MeM&T), Colegio de Ciencias de la Salud (COCSA), Escuela de Medicina, Edificio de Especialidades Médicas, Instituto de Simulación Computacional (ISC-USFQ), Universidad San Francisco de Quito (USFQ), Diego de Robles y vía Interoceánica, Quito 170157, Ecuador

**Keywords:** Cnidaria, antimicrobial, omics, artificial intelligence, complex networks

## Abstract

Overcoming the growing challenge of antimicrobial resistance (AMR), which affects millions of people worldwide, has driven attention for the exploration of marine-derived antimicrobial peptides (AMPs) for innovative solutions. Cnidarians, such as corals, sea anemones, and jellyfish, are a promising valuable resource of these bioactive peptides due to their robust innate immune systems yet are still poorly explored. Hence, we employed an in silico proteolysis strategy to search for novel AMPs from omics data of 111 Cnidaria species. Millions of peptides were retrieved and screened using shallow- and deep-learning models, prioritizing AMPs with a reduced toxicity and with a structural distinctiveness from characterized AMPs. After complex network analysis, a final dataset of 3130 Cnidaria singular non-haemolytic and non-toxic AMPs were identified. Such unique AMPs were mined for their putative antibacterial activity, revealing 20 favourable candidates for in vitro testing against important ESKAPEE pathogens, offering potential new avenues for antibiotic development.

## 1. Introduction

The global health crisis posed by antimicrobial resistance (AMR) led to an urgent need for novel therapeutic strategies. Antimicrobial peptides (AMPs), innate immune system components found across various species, offer a promising opportunity due to their multifaceted mechanisms and reduced susceptibility to resistance [1,2]. Unlike conventional antibiotics, which target intracellular pathways, AMPs also disrupt bacterial cell membranes, a vital structure not easily modified through mutations [3,4], reducing the risk of AMR development. Moreover, AMPs exhibit a broad spectrum of activities, including antibacterial, antifungal, antiviral, and antitumoral properties [2]. The structural diversity of the AMPs, distributed into three major structural classes (α-helices, β-sheets, and extended coils), support their distinct biological activities [5]. However, they generally share common structural features accounting for antimicrobial action, such as small size, positive charge, and amphiphilic characteristics, allowing them to interact particularly with the negatively charged membranes of microorganisms over the mammalian membranes [3,4,6,7]. To accelerate the development of AMPs as therapeutic agents, researchers have compiled comprehensive databases that have integrated information from various sources and analysis tools [8]. The StarPepDB [9], a graph database that integrates peptides and their metadata from 40 distinct AMP databases, stands out as one of the most complete curated repositories, addressing challenges related to data redundancy, duplication, and user interface. Other notable databases include DRAMP (Data Repository of Antimicrobial Peptides) [10] and DBAASP (Database of Antimicrobial Activity and Structure of Peptides) [11], which have been continuously updated to provide researchers with valuable resources.

Marine invertebrates, having evolved in challenging environments for more than 450 million years with high microbial and viral loads (about 10^6^ bacteria/mL and 10^9^ virus/mL of seawater [12]) and without adaptative immune responses, have developed robust innate immune systems that rely heavily on AMP production [4,6,13]. Hence, these animals have gained increasing attention as a source of AMPs, with unique structures and diverse antimicrobial activities, accounting for 67% of all marine-derived AMPs (statistics as of December 2022) [7,13]. AMPs have been identified in horseshoe crabs [14,15], polychaetas [16,17], mussels [18,19], ascidians [20,21], and sea urchins [22,23]. While AMPs have been isolated from numerous marine invertebrates, cnidarians–encompassing Anthozoa (corals and sea anemones) and Medusozoa (jellyfish and hydroids)—stand out as particularly promising organisms. To date, around 13 AMPs from this group have been thoroughly characterized (Table 1). With an estimated 13,300 species, these venomous animals possess nematocysts, specialized stinging cells loaded with toxins, which are used for both hunting and protection [24,25,26,27]. The pioneering isolation of Aurelin from the scyphoid jellyfish *Aurelia aurita* marked a significant breakthrough in cnidarian AMP research. This peptide, extracted from the mesoglea, demonstrated potent antimicrobial activity against both Gram-positive and Gram-negative bacteria, and its unique structure set it apart from other known AMPs [28]. Later, Damicornin, isolated from the stony coral *Pocillopora damicornis*, has exhibited both antibacterial and antifungal properties [29]. Intriguingly, some sea anemone’s neurotoxins also possess antimicrobial properties, suggesting a dual role in their survival. These toxins not only aid in capturing prey but also protect the animals from bacterial infections that may arise from tentacle damage [30,31].

Omics technologies have revolutionized our ability to discover novel bioactive peptides, including marine-derived AMPs. By analysing genomes, transcriptomes, and proteomes, researchers can identify potential AMPs based on their homology to known sequences using BLAST or Hidden Markov Models [32,33,34,35]. However, artificial intelligence (AI)-based algorithms are increasingly being used to uncover and predict the activity of these peptides, as demonstrated in one of our previous works with cephalopod posterior salivary glands [36]. Unlike homology-based methods, which rely on sequence similarity to known AMPs, AI-based approaches can identify novel peptides with unique structures and activities. These algorithms analyse large datasets of known AMPs sequences to identify essential physicochemical and sequence-based pattern determinants of AMP functionality. Then, by recognizing these patterns, they predict and rank the activity of new sequences, even those that may not have homology to previously characterized peptides [3,37].

With the pressing demand for innovative therapeutic agents to address AMR, this work focused on the exploration of omics data from cnidarians as a promising source of AMPs. We performed an in silico proteolysis-driven approach and employed machine-learning algorithms and complex network analysis to propose new marine-derived AMPs from these ancient organisms, including their putative activities and targets.

**Table 1:** Antimicrobial peptides (AMPs) identified from Cnidaria.

ID	Species	Subphylum	Uniprot	Length (AA)	Targets	Reference
Aurelin	*Aurelia aurita*	Medusozoa	Q0MWV8	84	Gram+ and Gram− Bacteria	[28]
AmAMP1	*Acropora millepora*	Anthozoa	P0DUG2	117	Gram+ and Gram− Bacteria	[38]
Arminin **	*Hydra vulgaris*	Medusozoa	D2XUU4	88	Gram+ and Gram− Bacteria	[39]
ATX-II *	*Anemonia sulcata*	Anthozoa	P01528	80	*Micrococcus luteus*	[31]
Crassicorin-I and Crassicorin-II *	*Urticina crassicornis*	Anthozoa	A0A1X9QHL1 and P0DUG3	79	*Bacillus subtilis, Escherichia coli* and *Salmonella enterica*	[30]
Damicornin	*Pocillopora damicornis*	Anthozoa	F1DFM9	107	Gram+ bacteria and the fungus *Fusarium oxysporum*	[29]
Equinin B	*Actinia equina*	Anthozoa	n.a.	72	*Escherichia coli*, *Micrococcus luteus* and *Vibrio alginolyticus*	[40]
Hydramacin-1	*Hydra vulgaris*	Medusozoa	B3RFR8	84	Gram+ and Gram− Bacteria	[41]
APETx1 *	*Anthopleura elegantissima*	Anthozoa	P61541	42	*Salmonella enterica*	[30]
ShK *	*Stichodactyla helianthus*	Anthozoa	P29187	35	*Bacillus subtilis, Escherichia coli, Salmonella enterica and Pseudomonas aeruginosa*	[30]
Kazal2	*Hydra magnipapillata*	Medusozoa	B8Y8I5	168	*Staphylococcus aureus*	[42]

* Indicates dual-functionality as both AMPs and toxins; ** Detected solely at transcriptomic level; AA—amino acids; n.a.—data not available.

## 2. Results

### 2.1. Cnidaria Databases Reveal Significant Uniqueness

**Figure 1:**
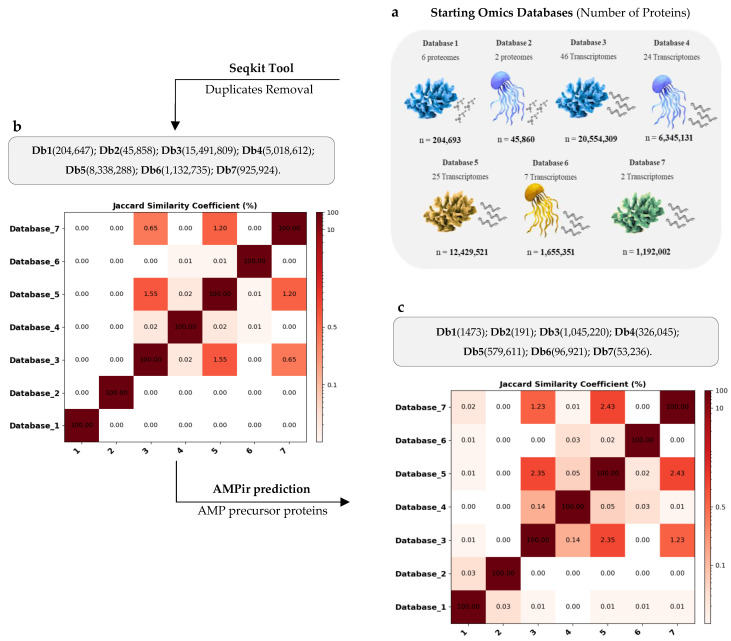
(**a**) Total protein counts across the omics databases. db1–db2 represent proteomes derived from genomic data, while db3–db7 include TSA and SRA transcriptomes (NCBI). Anthozoa are in db1, db3, db5, and db7, and Medusozoa are in db2, db4, and db6. Blue icons (db1–db4) are whole-body or non-specific samples, yellow (db5–db6) are tentacle samples, and green (db7) are nematocyst samples. (**b**) Jaccard similarity coefficient (%) showing protein diversity after duplicate removal using Seqkit. (**c**) Jaccard similarity coefficient (%) for the diversity of putative AMP precursor proteins, filtered by AMPir prediction (precursor model).

Seven protein databases (dbs) were constructed using data from eight proteomes derived from genomic data and 104 transcriptomes of 111 species of Cnidaria (Supplementary Table S1). Of these transcriptomes, 27 were sourced from SRA (Sequence Read Archive) data, and 77 were derived from TSA (Transcriptome Shotgun Assembly) data. The completeness scores for the 27 SRA-derived assembled transcriptomes ranged from 50.3% (SRR14115226) to 98.2% (SRR14115230), with 20 of the 27 transcriptomes (74.07%) exhibiting greater than 80% completeness (Supplementary Figure S1; Supplementary Table S2). TransDecoder was then employed to predict proteins across all transcriptomes (db3–db7). This led to db3 containing the highest number of proteins (20,554,309), followed by db5 (12,429,521), db4 (6,345,131), db6 (1,655,351), db7 (1,192,002), db1 (204,693), and db2 (45,860) (Figure 1a). After removing duplicates from each database, the total number of proteins decreased to the following: db1 (204,647), db2 (45,859), db3 (15,491,809), db4 (5,018,612), db5 (8,338,288), db6 (1,132,735), and db7 (925,924). All protein dbs displayed significant uniqueness, as measured by the Jaccard Index. The highest similarity coefficient was as small as 1.55% between db3 and db5 and 1.22% between db5 and db7, both representing Anthozoa (Figure 1b). The corresponding FASTA files for the non-duplicated protein libraries are available in Dataset 1. Subsequently, all protein dbs were processed with AMPir (precursor model) to predict their antimicrobial potential. After this prediction step, the number of proteins was reduced to: db1 (1473), db2 (191), db3 (1,045,220), db4 (326,045), db5 (579,611), db6 (96,921), and db7 (53,236). The same trend of uniqueness persisted after antimicrobial prediction, showing very low similarity coefficients, exceeding just 1% only in pairs db3–db5 (2.35%), db5–db7 (2.43%), and db3–db7 (1.23%) (Figure 1c). The FASTA files for the AMP precursor libraries, post-duplicate removal, and AMPir prediction are available in Dataset 2. Overall, the total number of non-redundant (nr) proteins was reduced from 25,264,871 to 1,939,076 nr AMP precursors (Supplementary Table S3). Each of these AMP precursor libraries was then used as input for the in silico proteolysis protocols to uncover novel AMPs.

### 2.2. In Silico Proteolysis of AMP Precursor Datasets with Distinct Proteases Yields Diverse Peptidomes

**Figure 2:**
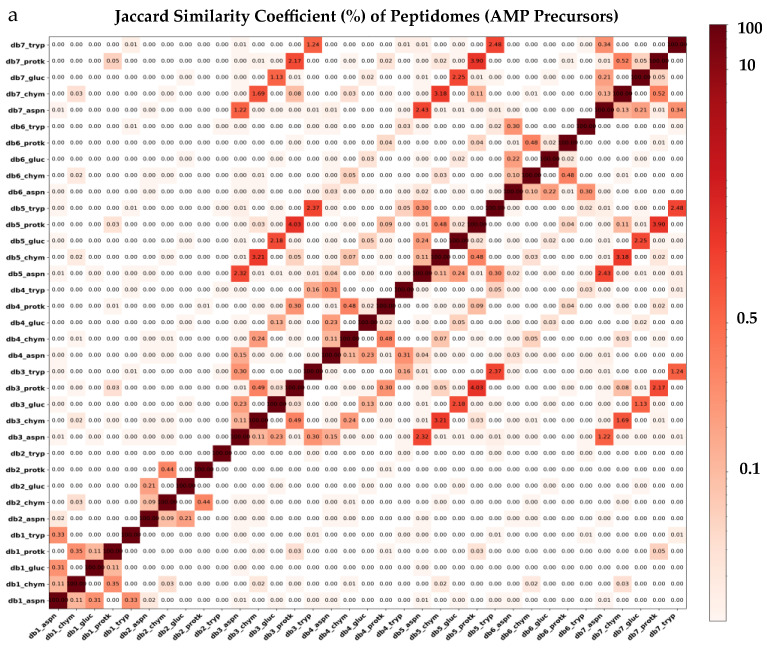

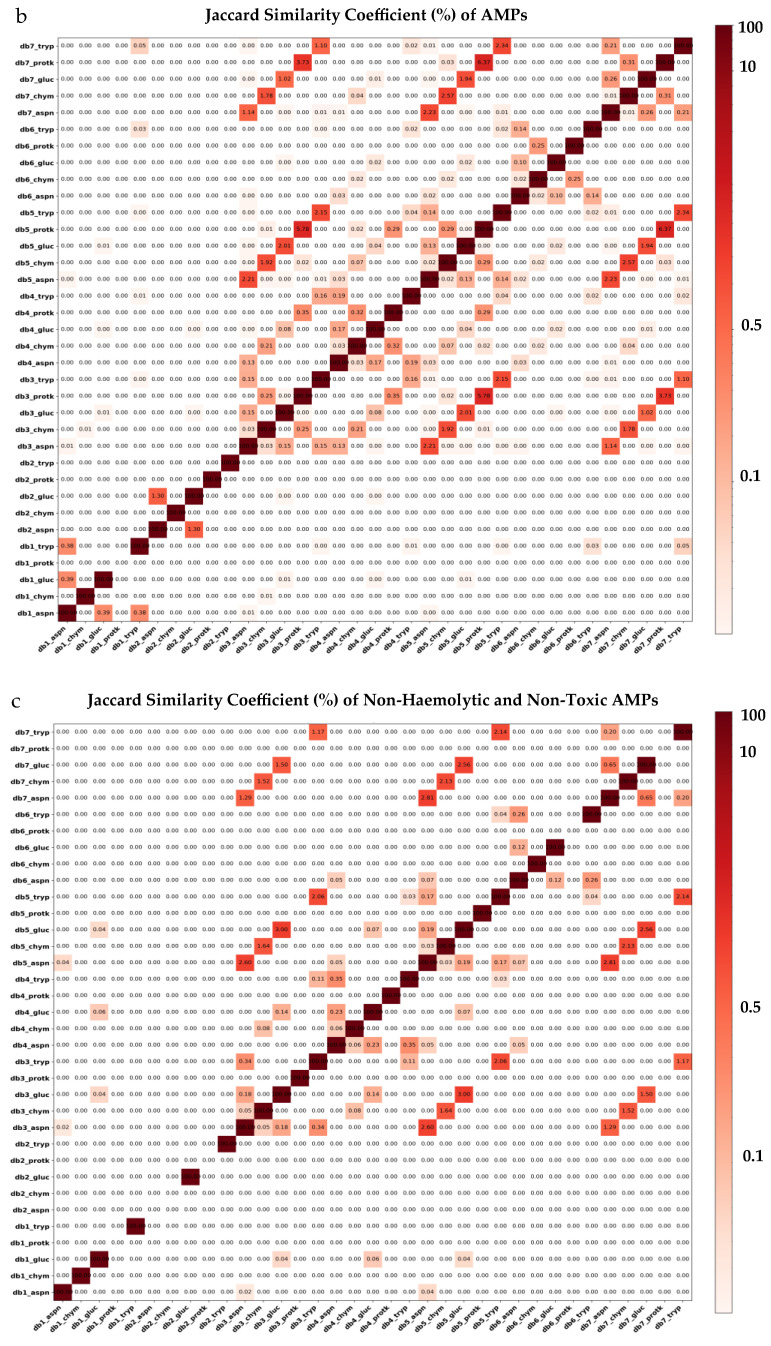
Peptide diversity across five proteolysis protocols in the seven Cnidaria databases during the AMP mining process, with the Jaccard similarity coefficient (%) used as a pairwise similarity metric to compare the 35 generated peptidomes. (**a**) Virtual peptidomes are generated by the in silico proteolysis protocol of the putative protein AMP precursors. (**b**) AMPs were identified through the consensus of three prediction models—AMPir, Macrel, and AMPlify—applied to the peptidomes from panel (**a**). (**c**) Non-haemolytic and non-toxic AMPs were identified through the consensus of Macrel, HemoPi, and MQSSM (for haemolytic predictions) and ToxinPred3, ToxTeller, and CAPTP (for toxic predictions), applied to the AMP libraries from panel (**b**).

After performing the in silico proteolysis using five distinct proteases—AspN, Chymotrypsin (Chym), GluC, Proteinase K (ProtK), and Trypsin (Tryp)—on the seven Cnidaria AMP precursors datasets, we generated 35 virtual peptide libraries, each corresponding to a specific protease–database combination. The resulting peptidomes were subsequently filtered to align with AMP characteristics by retaining peptides between 6–40 amino acids (AA) in length, removing duplicated and redundant peptides and eliminating peptides with non-standard AA (Table 2). The final peptides ranged from 11 to 40 AA in length, with an average length of 19 (Supplementary Table S4). After concatenating the peptide libraries from all protease–database combinations, we obtained a total of 12,428,038 peptides. Following redundancy reduction with cd-hit at a 0.98 sequence identity threshold, the dataset was reduced to 8,278,560 representative and nr peptides from AMP precursors. AspN and Trypsin produced the highest number of peptides in db1–db2 and db3–db7, respectively, while Protease K gathered the lowest number of peptides in all databases (Table 2). Db3 contains the highest number of nr peptides (*n* = 4,229,977), followed by db5 (*n* = 2,316,742), db4 (*n* = 1,357,350), db6 (*n* = 404,314), db7 (*n* = 230,192), db1 (*n* = 10,955), and db2 (*n* = 1788) (Table 2). The corresponding FASTA files for all the 35 peptide libraries after applying in silico enzymatic digestion protocols, along with the total concatenated datasets, are provided in Dataset 3. The peptidomes displayed a remarkable level of uniqueness, making them ideal for further antimicrobial and toxicity predictions. The maximum similarity coefficients, calculated using the Jaccard Index, were 4.03%, 3.90%, 3.21%, and 3.18%. These values corresponded to the pairs db3_protk/db5_protk, db5_protk/db7_protk, db3_chym/db5_chym, and db5_chym/db7_chym, respectively, all within the Anthozoa group (Figure 2a).

### 2.3. Antimicrobial and Toxicity Screening of Virtual Peptidomes Reveals High AMP Diversity

The 35 peptidomes generated from the previous in silico proteolysis were screened using a combination of three prediction tools to evaluate antimicrobial (AMPir, AMPlify, and Macrel), haemolytic (HemoPi, Macrel, and MQSSM), and toxic (CAPTP, ToxinPred3, and ToxTeller) properties, allowing for the final selection of non-haemolytic and non-toxic AMPs. This screening resulted in a total of 315 predictions. Detailed outputs from each prediction tool are provided in Dataset 4. To ensure accuracy, a consensus-based approach between three prediction tools in each mining step was employed. Venn diagrams were used to visualize the overlap between the predictions of different tools at each stage (Supplementary Figures S2–S4). This allowed for the identification of peptides that consistently met the desired criteria. After prediction, the datasets were filtered to remove redundant sequences and those that did not meet the specified criteria (e.g., haemolysis or toxicity). The final peptide datasets included 527,096 AMPs, 125,403 non-haemolytic AMPs, and 29,528 non-haemolytic and non-toxic AMPs. After reducing redundancy with CD-HIT at a 0.98 sequence identity threshold, these datasets were refined to 473,747 unique AMPs, 119,531 unique non-haemolytic AMPs, and 28,279 unique non-haemolytic and non-toxic AMPs (Table 3). The corresponding FASTA files for all the peptide libraries after antimicrobial and toxicity predictions, along with the total concatenated datasets, are available in Dataset 5 (AMPs), Dataset 6 (non-haemolytic AMPs), and Dataset 7 (non-haemolytic and non-toxic AMPs). From the highest to the lowest number, nr consensus peptides were detected at each screening step (AMP and non-haemolytic AMP, and non-haemolytic and non-toxic AMP), with db3 having the highest number (*n* = 244,894; *n* = 62,423; *n* = 14,816), followed by db5 (*n* = 127,952; *n* = 31,909; *n* = 7580), db4 (*n* = 77,857; *n* = 19,583; *n* = 4638), db6 (*n* = 22,683; *n* = 5585; *n* = 1237), db7 (*n* = 13,177; *n* = 3456; *n* = 867), db1 (*n* = 537; *n* = 133; *n* = 36), and db2 (*n* = 90; *n* = 24; *n* = 2), respectively (Table 3).

The peptidomes from AMPs and non-haemolytic and non-toxic AMPs continue to display a remarkable level of uniqueness. The maximum similarity coefficients, calculated using the Jaccard Index, were 6.37% (db5_protk/db7_protk), 5.78% (db3_protk/db5_protk), 3.73% (db3_protk/db7_protk), and 2.57% (db5_chym/db7_chym) for AMPs; and 3.00% (db3_gluc/db5_gluc), 2.81% (db5_aspn/db7_aspn), 2.60% (db3_aspn/db5_aspn), and 2.56% (db5_gluc/db7_gluc) for non-haemolytic and non-toxic AMPs (Figure 2b,c). The final robust dataset of non-redundant, non-haemolytic, and non-toxic AMPs from Cnidaria, comprising 28,279 peptides, was selected for further analysis. These peptides offer considerable potential for biomedical research and drug development. Additionally, we provide specialized datasets tailored to various research needs.

### 2.4. Physicochemical Properties Indicate the Suitability of the Non-Haemolytic and Non-Toxic AMPs for Targeting Microbial Membranes

**Figure 3:**
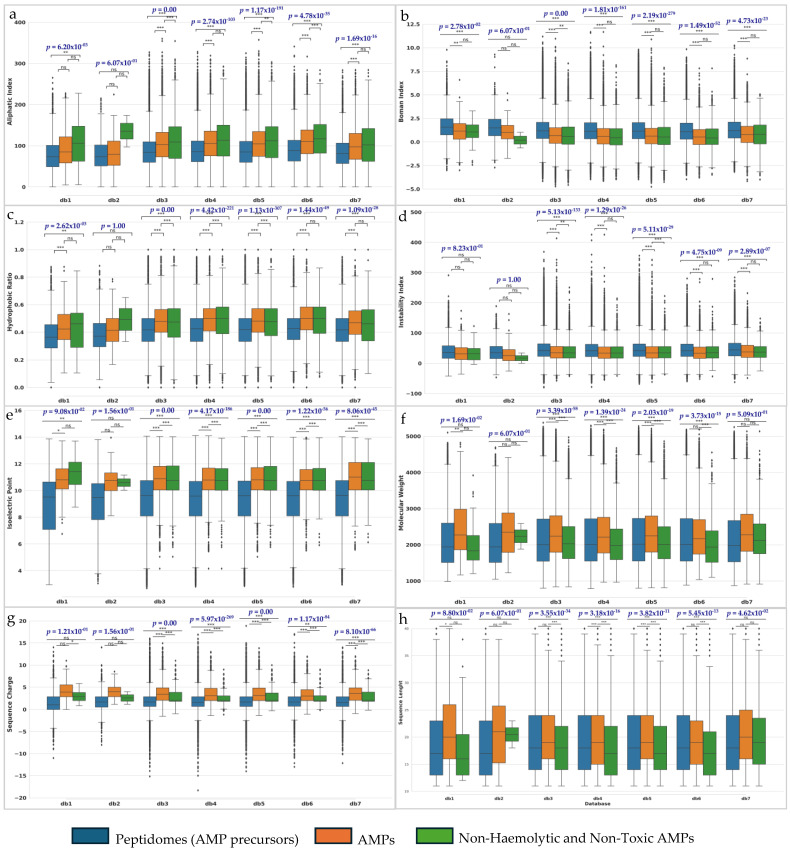
Physicochemical properties of the peptides generated by the in silico proteolysis of the putative protein AMP precursors (blue box); mature predicted AMP peptides (orange) and predicted non-haemolytic and non-toxic AMPs (green). The properties assessed include (**a**) aliphatic index; (**b**) Boman index; (**c**) hydrophobic ratio; (**d**) instability index; (**e**) isoelectric point; (**f**) molecular weight; (**g**); sequence charge; and (**h**) sequence length. Median values are represented with a trace. The highlighted *p* values indicate results from the Friedman test with truncated data, where statistical significance was defined as *p* < 0.05. Pairwise comparisons were conducted using Wilcoxon Signed-Rank tests adjusted with Bonferroni corrections, where * *p* < 0.05; ** *p* < 0.01; *** *p* < 0.001; and ns—not significant.

Shifts in global peptide characteristics are expected throughout the AMP mining process, particularly between the enzymatically processed AMP precursors, mature AMPs, and non-haemolytic and non-toxic AMPs. Essentially, significant differences (*p* < 0.05) for the variation of the physicochemical properties were found within all databases, except for db1 in sequence charge (*p* = 0.12), instability index (*p* = 0.82), isoelectric point (*p* = 0.09), and sequence length (*p* = 0.08); db2 in all properties (*p* > 0.05); and db7 in molecular weight (*p* = 0.51) (Figure 3). Then, Wilcoxon Signed-Rank tests were used to specifically identify significance in property variations by performing pairwise comparisons between the peptides, AMPs, and non-toxic AMPs (Figure 3) (here in the text, *p* values represent the differences between the peptides and the non-haemolytic and non-toxic AMPs). The aliphatic index, which reflects peptide thermostability and resistance to proteolytic enzymes, is elevated in AMPs and non-haemolytic and non-toxic AMPs, with significant differences between peptides and the final AMP dataset (db1: *p* = 0.001; db3: *p* = 0.0; db4: *p* = 1.70 × 10^−102^; db5: *p* = 9.18 × 10^−187^; db6: *p* = 1.40 × 10^−44^; db7: *p* = 2.19 × 10^−16^). Similarly, higher sequence charge (db3: *p* = 1.62 × 10^−78^; db4: *p* = 4.50 × 10^−06^; db5: *p* = 1.23 × 10^−45^; db6: *p* = 0.001; db7: *p* = 1.30 × 10^−15^) and isoelectric point (db1: *p* = 0.003; db3: *p* = 2.27 × 10^−161^; db4: *p* = 1.34 × 10^−69^; db5: *p* = 4.53 × 10^−163^; db6: *p* = 6.23 × 10^−21^; db7: *p* = 6.19 × 10^−24^) in non-haemolytic and non-toxic AMPs enhance their putative interaction with negatively charged microbial membranes. An increased hydrophobic ratio (db1: *p* = 0.008; db3: *p* = 0.0; db4: *p* = 6.38 × 10^−147^; db5: *p* = 1.20 × 10^−203^; db6: *p* = 1.43 × 10^−40^; db7: *p* = 2.47 × 10^−21^) also favours targeted interactions with microbial membranes over mammalian ones. While the median of molecular weight (db3: *p* = 1.20 × 10^−31^; db4: *p* = 5.69 × 10^−37^; db5: *p* = 1.77 × 10^−31^; db6: *p* = 2.89 × 10^−16^) and sequence charge (db3: *p* = 1.62 × 10^−78^; db4: *p* = 4.50 × 10^−06^; db5: *p* = 1.24 × 10^−45^; db6: *p* = 0.001; db7: *p* = 1.30 × 10^−15^) of AMPs are slightly higher than those of the precursors, they slightly decrease in non-haemolytic and non-toxic AMPs. This reduction benefits diffusion to microbial membranes and facilitates rapid antimicrobial activity. Conversely, the instability index shows minimal variation, but lower median values in AMPs and non-haemolytic and non-toxic AMPs suggest greater stability, albeit with slightly reduced flexibility (db3: *p* = 4.64 × 10^−136^; db4: *p* = 7.59 × 10^−24^; db5: *p* = 1.46 × 10^−15^; db6: *p* = 1.35 × 10^−08^; db7: *p* = 3.49 × 10^−07^). Additionally, the Boman Index median, which measures the potential for protein–protein interactions, is slightly lower in both AMPs and non-haemolytic and non-toxic AMPs (db1: *p* = 0.0004; db3: *p* = 0.0; db4: *p* = 2.28 × 10^−152^; db5: *p* = 9.66 × 10^−250^; db6: *p* = 2.55 × 10^−50^; db7: *p* = 3.94 × 10^−22^). This implies fewer interactions with host proteins, making these peptides less toxic and more specific to microbial membranes. Overall, these characteristics align with typical AMP properties, contributing to their antimicrobial efficacy and selectivity (Figure 3).

### 2.5. Cnidaria Singular AMPs (CnSAs) Demonstrate High Internal Sequence Diversity

**Figure 4:**
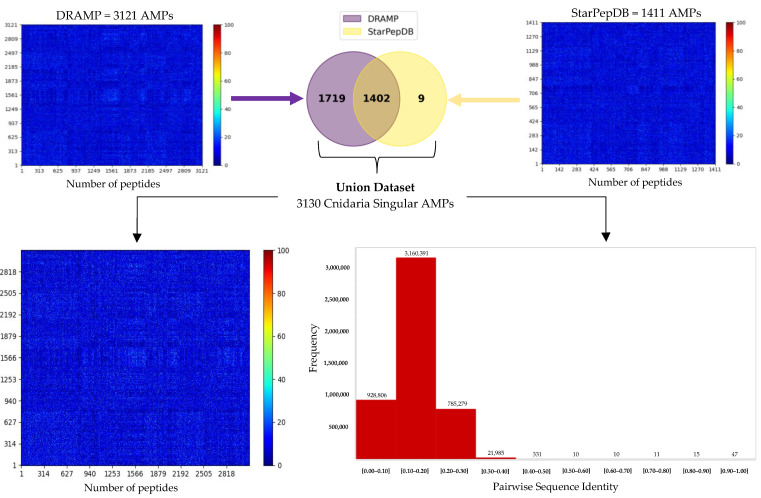
Heatmaps of the pairwise sequence identity of the 3121 and 1411 non-haemolytic and non-toxic AMPs were obtained after comparison with the publicly available AMP databases DRAMP and StarPepDB, respectively, with a sequence identity cutoff of 0.40 using CD-HIT-2D. The union of both datasets was selected, which represents the 3130 Cnidaria Singular AMPs (CnSAs). The heatmap and histogram of the pairwise sequence identity of the CnSAs were constructed, demonstrating similarities generally below 30%.

The singularity of the 28,279 non-haemolytic and non-toxic AMPs from Cnidaria was evaluated against 4951 and 19,456 non-redundant AMPs from the DRAMP and StarPepDB databases, respectively, using a sequence identity cutoff of 40%. Essentially, all sequences that have below 40% sequence identity with the public databases were retained. Among the 28,279 Cnidaria AMPs, 25,158 and 26,868 were found to cluster with members of DRAMP and StarPepDB, respectively, indicating a strong relationship with the known chemical space of characterized AMPs in both databases. The remaining 3121 and 1411 AMPs were subjected to further comparison, leading to the identification of 3130 Cnidaria Singular AMPs (CnSAs) through the union of both datasets (Figure 4). To assess the internal sequence diversity of the 3130 CnSAs—those sharing less than 40% similarity with both DRAMP and StarPepDB—a series of all-vs-all global alignments were performed (Figure 4). The results reveal a low sequence identity among most peptide pairs, with similarities generally below 30%. This pronounced singularity among the identified AMPs is crucial for their diverse antimicrobial activities. The FASTA files corresponding to the 3130 CnSAs, as well as the nr DRAMP and StarPepDB databases and the retrieved sequences with less than 40% with both databases, are accessible at Dataset 8.

### 2.6. Half-Space Proximal Networks (HSPNs) Facilitate the Extraction of Representative CnSA Datasets

**Figure 5:**
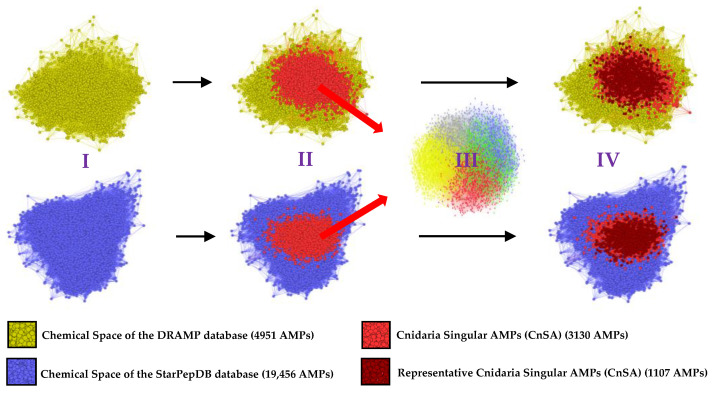
Workflow of the chemical space analysis using Half-Space Proximal Networks (HSPN) to uncover Cnidaria Singular AMPs (CnSAs). **I**: Visualization of the chemical spaces of DRAMP (yellow; 4951 AMPs) and StarPepDB (blue; 19,456 AMPs) databases. **II**: Superimposition of the CnSAs (light red; 3130 AMPs) on the chemical spaces of DRAMP and StarPepDB databases, separately; **III**: Representation of the HSPN of the extracted 3130 CnSA, demonstrating the presence of five distinct clusters. **IV**: Superimposition of the representative CnSA (dark red; 1107 AMPs) on the chemical spaces of DRAMP and StarPepDB databases, separately, which is centralized over the CnSA.

**Figure 6:**
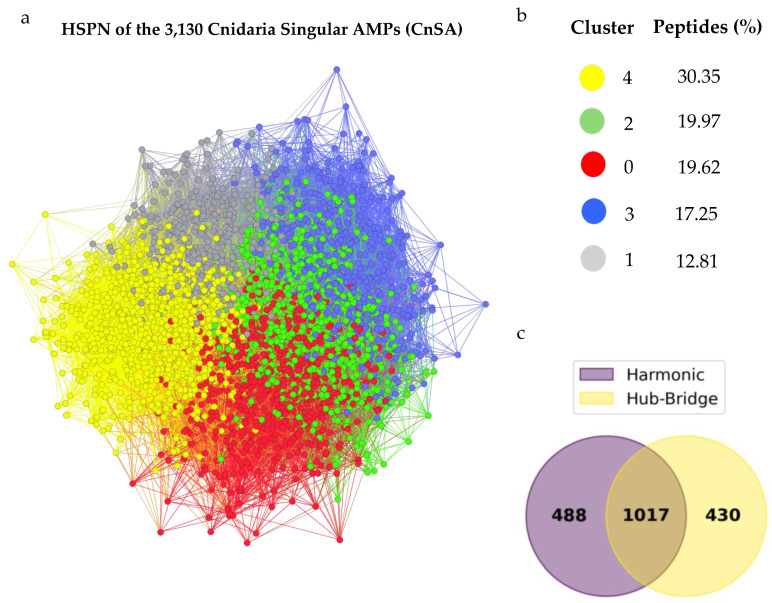
(**a**) Half-Space Proximal Network (HSPN) clustering of the 3130 non-haemolytic and non-toxic CnSAs, with clusters distinguished by colour. (**b**) Distribution of the CnSAs across the identified clusters. (**c**) Venn diagram illustrating the union and intersection of CnSA datasets from Harmonic centrality (1505 peptides) and Hub-Bridge centrality (1447 peptides) analyses. The resulting datasets include 1935 peptides (union) and 1017 peptides (intersection).

The chemical space of the CnSAs is superimposed over the chemical spaces of the DRAMP and StarPepDB databases by projecting a composite HSPN, made up of the three datasets (Figure 5). This analysis suggests that CnSAs occupy a different chemical space, concentrated in the red zone. These CnSAs have five distinct clusters, suggesting they may have distinct physicochemical properties and actions against microbial cells (Figure 6a). Cluster 4 contains the highest number of peptides (30.35%), followed by Cluster 2 (19.97%), Cluster 0 (19.62%), Cluster 3 (17.25%), and Cluster 1 (12.81%) (Figure 6b). To detect the most representative CnSA from the HSPN, we applied the harmonic (HC) and hub-bridge (HB) centrality measures. These measures of centrality are very popular in network science for the detection of relevance along all networks and within their clusters, respectively. This analysis resulted in the extraction of 1505 and 1447 representative AMPs from the 3310 CnSAs, respectively. The union and intersection of the two centrality-based subsets generated 1935 and 1017 unique AMPs, respectively (Figure 6c). Again, the chemical space of the intersection subset of the most representative CnSA is superimposed over the chemical spaces of the DRAMP and StarPepDB databases, confirming its representativeness when centralized over the CnSAs (Figure 5).

**Figure 7:**
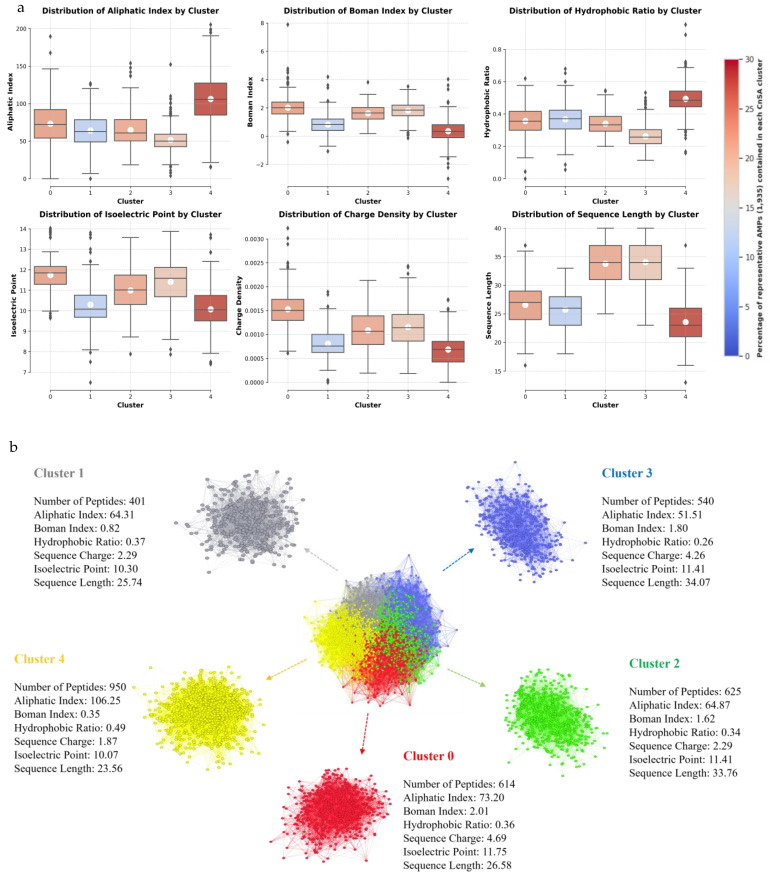
(**a**) Physicochemical characterization of the Cnidaria Singular AMPs (CnSA) clusters within the Half-Space Proximal Network (HSPN) chemical space. Boxplot colours indicate the percentage of representative AMPs—derived from the union dataset (1935 peptides)—in each cluster. Traces represent median values, while white circles denote mean values for the physicochemical properties within each cluster. (**b**) Number of peptides per cluster, alongside the mean values for each physicochemical property across the clusters.

The characterization of the CnSAs within the HSPN is provided in Supplementary File S5, detailing properties for each of the 3310 nodes and the HC and HB values. An HSPN was also constructed to represent the chemical space of the intersection dataset (1017) of the most representative CnSA, retaining the same five clusters of the previously mentioned space, depicted in Figure 6a However, their peptide content varies. Cluster 4 contains the highest number of peptides (23.21%), followed by Cluster 2 (22.42%), Cluster 3 (20.55%), Cluster 0 (19.37%), and Cluster 1 (14.45%) (Supplementary Figure S5). The FASTA files corresponding to the CnSA extracted via harmonic and hub-bridge network centralities, as well as for the union and intersection datasets, are available in Dataset 9. We considered the union dataset (1955) as the representative and comprehensive AMP dataset for further analysis. Cluster 4 has the highest percentage of representative AMPs (27.18%), followed by Cluster 0 (21.91%), Cluster 2 (21.14%), Cluster 3 (18.14%), and Cluster 1 (11.63%) (Figure 7a). The mean values for each characteristic are represented in Figure 7b.

### 2.7. Strain-Specific Predictions Reveal Novel Candidate Antibacterial Peptides (ABPs)

**Figure 8:**
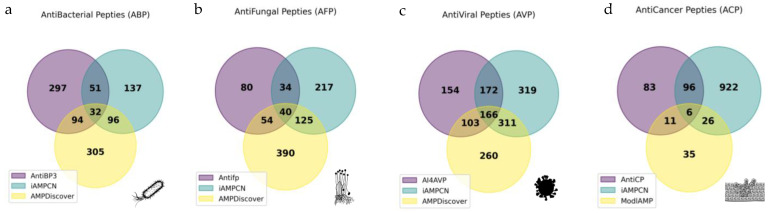
Predictions of the specific activities of non-haemolytic and non-toxic AMPs were obtained from the union dataset (1935) of the most representative Cnidaria AMPs. Three predictive tools were applied, generating both intersection and union datasets for each predicted activity type: (**a**) Antibacterial peptides (ABP); (**b**) Antifungal peptides (AFP); (**c**) Antiviral peptides (AVP); and (**d**) Anticancer peptides (ACP).

The union dataset of the 1935 Cnidaria AMPs obtained after centrality analysis was used for activity predictions of antibacterial (ABP), antiviral (AVP), antifungal (AFP), and anticancer (ACP) peptides. For the ABPs, antibacterial activity predictions were performed using three prediction tools, resulting in intersection and union datasets of 32 and 1012 candidate ABPs, respectively (Figure 8a). Besides ABPs, robust predictions for antifungal, antiviral, and anticancer activities were also retrieved, resulting in intersection datasets of 40; 166 and 6; and union datasets of 940, 1485, and 1179 for AFPs, AVPs, and ACPs, respectively (Figure 8b–d). The intersection and union datasets of ABPs were virtually tested against five distinct bacterial strains, demonstrating similar patterns between intersection and union candidates (Supplementary Figure S6a). After ranking the peptides (from I to IV, see methods Section 4.6) (Supplementary Figure S6b), a total of 152 ABPs were selected for further analysis. We obtained 5 peptides from Rank I, 8 from Rank II, 7 from Rank III, and 132 from Rank IV.

**Figure 9:**
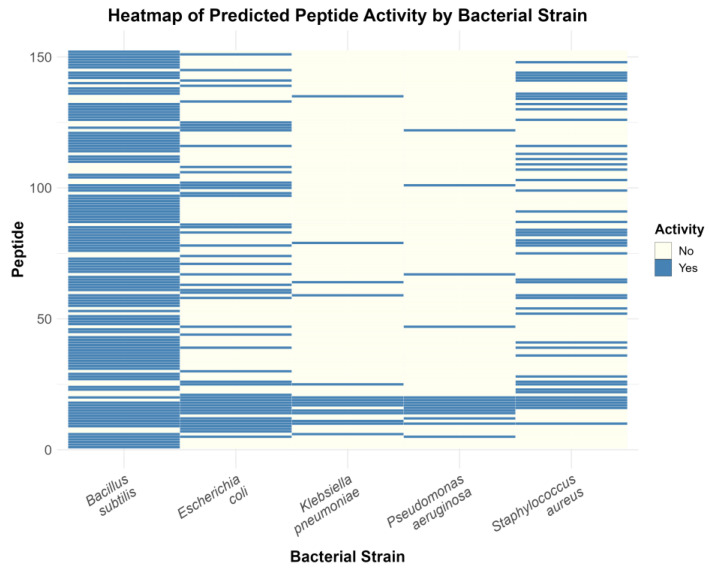
Distribution of the 152 antibacterial peptides (ABPs) from Rank I to IV across five bacterial strains identified as susceptible to inhibition by these peptides.

The sea anemones *Anthopleura elegantissima* and *Exaiptasia diaphana* bearded the highest number of ABPs (*n* = 8), followed by the stony coral *Alveopora japonica* (*n* = 7) (Supplementary Figure S7a). Also, db3 retrieved the highest number of ABPs (*n* = 91), followed by db5 (*n* = 31), db4 (*n* = 18), db6 (*n* = 9), and db7 (*n* = 3) (Supplementary Figure S7b). From these peptides, 77.63% (118/152) had predicted antibacterial activity against *Bacillus subtilis*, 32.24% (49/152) against *Escherichia coli*, 9.21% (14/152) against *Klebsiella pneumoniae*, 9.21% (14/152) against *Pseudomonas aeruginosa*, and 30.92% (47/152) against *Staphylococcus aureus* (Figure 9). Although the majority of ABPs were retrieved from Whole Body/Non-Specific tissues (*n* = 107), most ABPs were retrieved from tentacles (*n* = 42), with some being retrieved also from nematocysts (*n* = 3) (Supplementary Figure S7c). Additionally, only AspN (*n* = 94), GluC (*n* = 56) and Chymotrypsin (*n* = 2) produced ABPs. The peptides generated with Trypsin and Protease K showed no robust predicted activity against bacteria (Supplementary Figure S7d). Hexacorallia contributed to the highest number of ABPs (*n* = 109), with stony corals (Scleractinia) and sea anemones (Actiniaria) contributing most to 53/109 and 42/109 ABPs, respectively, followed by Octocorallia (*n* = 16), Hydrozoa (*n* = 12), Scyphozoa (*n* = 9), Staurozoa (*n* = 3), and Cubozoa (*n* = 3) (Supplementary Figure S7e). All the information regarding the 152 ABPs is available in Supplementary Table S6. Detailed outputs from each prediction tool are provided in Dataset 10. The FASTA files corresponding to the predicted intersection and union datasets of ABPs, AFPs, AVPs, and ACPs are available in Dataset 11. For the ABPs, we considered the Rank I, II, and III peptides more promising for further testing. They consist of 20 ABPs, 19 from Anthozoa and 1 from Medusozoa. The AA sequences from these 20 ABP candidates are available in Table 4. All peptides from Rank I demonstrate putative activity against *Bacillus subtilis*, with one from the stony coral *Fimbriaphyllia ancora* also demonstrating activity against *Escherichia coli* and *Pseudomonas aeruginosa*. Rank II AMPs demonstrate putative activity against 1one-to-five strains, while Rank III AMPs demonstrate putative activity against four-to-five strains. The FASTA files corresponding to Rank I, Rank II, Rank III, and Rank IV ABPs are available in Dataset 12. Additionally, strain-specific predictions of AFPs and AVPs (Supplementary Figure S8), as well as specific targets of ACPs, are available at Dataset 12.

## 3. Discussion

The concept of in silico research emerged in the late ‘90s, revolutionizing how biological experiments could be conducted. Over time, it has become an indispensable tool for preliminary screening, prediction, and optimization in biological research [43]. By cutting off the data processing time and reducing costs, in silico methods, currently reinforced by AI, allow researchers to narrow down vast protein data repositories and focus on promising leading compounds for further experimental validation. This in silico biodiscovery project, along with other related ones [8,44], including the recent discovery of AMPs in omics data of cephalopod salivary glands [36] and prediction of tumour-homing peptides in AMPs from public databases [45], are prime examples where the achieved results would not have been feasible through in vitro analysis alone.

Here, we incorporated seven distinct omics datasets from 111 species of Cnidaria to increase the likelihood of identifying novel AMPs with promising activities, as these are typically encoded by species-restricted genes [46]. In other work using omics data, candidate AMPs have been identified through transcriptomic analysis of the fire coral *Millepora complanata* [47], and defensine-like AMPs have been uncovered in several Cnidaria genomes [35] and transcriptomes [34]. In this work, we combined data from whole-body tissues and tentacles, which are more likely to interact with microbes due to tentacle damage during prey capture or defence [30,31]. We also used two transcriptomes from nematocysts, which may contain a mixture of bioactive molecules, including toxins and AMPs, as demonstrated by the antimicrobial activity of cnidocysts isolated from polyps of the zoanthid *Parazoanthus axinellae* against human pathogens [48]. We started with a vast quantity of nr distinct proteins (25,264,871), which were further reduced by applying the precursor model of AMPir (1,939,076) [49]. We found this method effective for narrowing the focus to precursor proteins that may result in more AMPs while still retaining a strong dataset.

To replace the laborious proteomics protocols, advanced tools like RPG v2.0.5 [50] efficiently generate diverse encrypted peptides hidden in protein sequences by simulating protease-induced cleavage. The use of different virtual proteases in this work contributed to the high diversity of the resulting peptidomes, which showed low similarity between them, accounting for a total of nr 8,278,560 peptides. This is because trypsin generates short-length peptides with a basic Arg or Lys at the C-terminus [51]; chymotrypsin targets aromatic AAs [52]; proteinase K does not rely on specific cleavage motifs (abroad specificity) [53]; and AspN and GluC target acidic AA [36].

By applying three distinct prediction methods to evaluate antimicrobial, haemolytic, and toxicity properties—drawing inspiration from our previous study on cephalopod salivary glands [36]—we improved the accuracy and reliability of identifying AMPs that are both non-haemolytic and non-toxic to red blood cells and mammalian cells, respectively, thus removing the most undesired characteristics of AMPs [54,55] and ensuring their suitability for future applications. Each tool utilizes different algorithms, prediction models, and features, complementing one another in identifying potential AMPs. Of the 473,747 AMPs, only 28,279 were predicted as non-haemolytic and non-toxic, revealing that not all AMPs obtained from marine organisms are safe to use. All databases were rather unique, as reflected by the Jaccard similarity coefficient. While sequence similarity is a valuable initial assessment to clarify overall similarities, it does not inherently imply functional or structural equivalence, particularly for peptides with fewer AA sequences. Nevertheless, for our purposes, it proved to be an effective and computationally efficient method for assessing similarities across large databases. A previous study on the transcriptomes of tentacle secretions of the sea anemone *Cnidopus japonicus* also used similar algorithms to predict antimicrobial and cytotoxicity properties of AMPs within toxin-like proteins. Later, some demonstrated in vitro antimicrobial activity against *B. subtilis* and *E. coli* [56].

Indeed, our analysis revealed that the characteristics of the final set of non-haemolytic and non-toxic AMPs closely resemble those of typical AMPs, indicating that our mining process was successful in identifying peptides with expected functional properties. As anticipated, several key features—such as the aliphatic index, hydrophobic ratio, isoelectric point, and sequence charge—exhibit higher median values for AMPs and non-haemolytic and non-toxic AMPs compared to their precursor forms. Additionally, these AMPs display a balanced Boman index and instability index, along with shorter sequence lengths and lower molecular weights.

The primary reason we compared our predicted AMPs to DRAMP and StarPepDB was to ensure the identification of novel AMP scaffolds, which were reduced to 3130 CnSAs. By using complex network analysis, they formed five clusters exhibiting distinct physicochemical properties, suggesting potential differences in their functionalities. Each cluster may contain peptides tailored to specific antimicrobial roles or interactions within the host organism’s immune system. Also, the size of these clusters is proportional to the content of the representative peptides obtained after centrality analysis (1935; union dataset), indicating that the reduction process, achieved through network-based rules, was effective.

Finally, specific activities for our final AMP dataset were predicted by following the same strategy as before. The three prediction tools were applied for antibacterial, antiviral antifungal, and anticancer peptides, as well as strain/target-specific predictions. We provided all the datasets for researchers to test their activity in vitro. Due to the availability of three strain-specific prediction tools in DBSAASP [11] for ABPs, this allowed for a more thorough evaluation, where we ranked these peptides considering the robustness of the predictions driven by the intersection and union of the evaluated models. The predictions over multidrug-resistance ESKAPEE pathogens, which are major contributors to antibiotic resistance and healthcare-associated infections, were also prioritized [57]. This led to the proposal of 152 ABPs from Cnidaria, with 20 candidate ABPs with the potential to be tested in vitro. The higher availability of omics data from Anthozoa leads to a higher prediction of ABPs from this group. Also, the usage of tentacle samples was ideal as it accounted for 42/142 of the total ABPs predicted, and although we only used two transcriptomes from nematocysts, three peptides were still uncovered. AspN and GluC retrieved a higher amount of ABPs, while Trypsin did not, revealing the importance of using distinct proteases. Although most peptides displayed predicted activity against *B. subtilis*, it is necessary to prioritize the activity against the other ESKAPEE pathogens. In previous works, AMPs originating from cDNA expression (EST) libraries from the jellyfish *Aurelia aurita* and the Ctenophore comb jelly *Mnemiopsis leidyi* demonstrated antibiofilm properties [58], and four synthetic AMPs predicted from the transcriptome of *Hydractinia symbiolongicarpus* were active against both Gram-positive and Gram-negative bacteria (including *P. aeruginosa* and *S. aureus*), with little-to-no haemolytic effects [59]. So, our 20 candidate ABPs may be potentially active, and we strongly recommend them for in vitro testing.

In this work, we show how digital solutions can streamline a massive dataset of 25,264,871 proteins into 1935 representatives of Cnidaria Singular AMPs (CnSA) and 20 candidate ABPs, which passed all stages of our rigorous mining process. We provide detailed datasets designed for various research needs, from broad-spectrum antimicrobial studies to strain-specific experimental validations. This approach can be applied not only to Cnidaria omics data but also to other marine invertebrates. Some limitations of this study included the reliance on publicly available omics data, which are biased towards Anthozoans. Additional handicaps may stem from the fact that some prediction tools may be more accurate than others, with some highly cited by the literature (ex: Macrel, AMPir, HemoPi and ToxinPred3) and some more recently published (ex: ToxTeller and CAPTP), although it may not be an indication of higher reliability. That is why we only considered the commonly predicted AMPs. One possible solution may be to experimentally validate intermediate or final predicted peptides to clarify the accuracy of such tools. Nevertheless, studies like this play a crucial role in uncovering novel AMPs from unexplored marine invertebrates and in advancing the field of antimicrobial research.

## 4. Materials and Methods

### 4.1. Gathering of Omics Data from Cnidaria

A total of 8 proteomes derived from genomic data and 104 transcriptomes from 111 species of Cnidaria were collected, encompassing 78 Anthozoa and 33 Medusozoa (Supplementary Table S1). These transcriptomes were obtained from diverse tissues—whole body/non-specific (*n* = 70), tentacles (*n* = 32), and nematocysts (*n* = 2). Notably, the sea anemone *Heterodactyla hemprichii* had data available from both tentacles and nematocysts. Proteomic data were obtained from the UniProt Proteome Database (https://www.uniprot.org/, accessed on 5 January 2024). Transcriptomes were retrieved from the Sequence Read Archive (SRA) and Transcriptome Shotgun Assembly (TSA) databases at the National Centre for Biotechnology Information (NCBI) (https://www.ncbi.nlm.nih.gov/, accessed on 5 January 2024). FASTq files associated with each SRA entry were then downloaded through the European Nucleotide Archive (https://www.ebi.ac.uk/ena/browser/, accessed on 5 January 2024). To ensure high-quality data, we employed Trimmomatic v0.39 [60] for read trimming and adapter removal, followed by quality control with FastQC v0.12.0 [61]. De novo transcriptome assembly was performed for both single-end and paired-end reads using Trinity v2.15.1 [62]. Finally, BUSCO v5.5.0 [63] assessed assembly completeness using the Metazoa lineage (Supplementary Table S2).

### 4.2. Database Construction

To identify coding regions within the transcriptomic data, we used TransDecoder v5.7.1 (https://github.com/TransDecoder/TransDecoder, accessed on 8 January 2024) with a minimum open reading frame of 50 AA. We then constructed 7 protein databases (db) categorized by species and tissue type (whole body/non-specific, tentacles and nematocysts) for both Anthozoa and Medusozoa: Db1—6 proteomes derived from sequenced genomes of Anthozoa; Db2—2 proteomes derived from sequenced genomes of Medusozoa; Db3—46 whole-body/non-specific transcriptomes of Anthozoa; Db4—24 whole-body/non-specific transcriptomes of Medusozoa; Db5—25 transcriptomes specific to the tentacles of Anthozoa; Db6—7 transcriptomes specific to the tentacles of Medusozoa; Db7—2 transcriptomes specific to the nematocysts of Anthozoa. Duplicate sequences within each db were removed using Seqkit tool v2.6.1 [64]. Since AMPs often originate from larger precursor proteins, we screened all dbs for potential antimicrobial proteins using the AMPir (antimicrobial peptide prediction in r) precursor model [49]. Following this, the Seqkit tool v2.6.1 [64] was again employed to evaluate sequence similarities within and between the dbs before and after AMP screening. The Jaccard Index, expressed as percentages, served as the metric for all-vs-all pairwise similarity comparisons among databases [65] to evaluate their overall compositional similarities in amino acid content.

### 4.3. In Silico Proteolysis

To generate individual peptidomes from each protein db displaying antimicrobial potential, identified with AMPir, we performed single-enzyme proteolysis using five commonly employed enzymes in proteomics (AspN, Chymotrypsin, GluC, Proteinase K, and Trypsin) with RapidPeptidesGenerator (RPG) v2.0.5 [50]. This resulted in a total of 35 individual peptidomes. Subsequently, we applied a series of filtering steps to each peptidome, including retaining peptides between 6 and 40 AA in length, removing duplicate peptides, eliminating peptides sharing above 98% of sequence identity, and excluding peptides containing non-standard AA. This filtering process used both the Seqkit tool v2.6.1 [64] and the CD-HIT tool v4.8.1 [66], ensuring that the final peptidomes contained high-quality peptides suitable for further analysis.

### 4.4. Antimicrobial and Toxicity Screening

To identify promising AMPs within each peptidome, we screened for those with no haemolytic activity and no toxic signatures. We adopted a consensus approach, considering peptides commonly predicted by three of the following models: Antimicrobial activity: (i) the alternative/mature model from AMPir [49], best suited for sequences after post-translational processing; (ii) Macrel: (Meta)genomic AMP Classification and Retrieval [67], by running the subcommand “macrel peptides”; and (ii) AMPlify [68], a deep-learning (DL) model for AMP prediction. Each one of these tools uses different classifiers—Support Vector Machine (SVM), Random Forest (RF), and Deep Neural Networks (DNN), respectively, maximizing the discovery of novel AMPs. Peptides with a probability of antimicrobial activity greater than 0.5 across all tools were selected. Haemolytic potential: (i) Macrel [67], as the output of the subcommand “macrel peptides”, also contains haemolytic probability, while the “NonHemo” AMPs were selected”; (ii) the hybrid model in the standalone version of HemoPi [69], which integrates motif-based and SVM-based predictions, was used to considered to identify peptides with a score of <0.5 as non-haemolytic; and (iii) a multi-query similarity searching model (MQSSM-I1) developed by [70], which can retrieve haemolytic peptides from a representative subset of the haemolytic sequence space. Consequently, the non-identified hits were treated as non-haemolytic peptides for our analysis. Toxic signatures: (i) a hybrid model implemented in ToxinPred3 [71], which combines motif- and ML-based predictions, where peptides with a recommended score of <0.38 were considered as non-toxic; (ii) ToxTeller [72], which contains four different ML-based predictors using logistic regression (LR), SVM, RF, and a scalable end-to-end tree-boosting system (XGboost), where we selected only the peptides predicted with a probability of 0 (non-toxic) among all models; and (iii) CAPTP [73], which uses in silico mutagenesis (ISM) interpretation methods, where we selected only those AMPs predicted as “non-toxic”. The consensus predictions among of all the outputs generated at each screening step were identified through the construction of Venn Diagrams (Supplementary Figures S2–S4). Using Seqkit tool v2.6.1 [64], we conducted an all-vs-all comparison of the 35 generated dbs at each mining step (1-peptidomes, 2-AMPs, and 3-Non-haemolytic/non-toxic AMPs), employing the Jaccard Index as a pairwise similarity metric [65]. Then, to explore differences between peptidomes and AMP-based peptidomes, we analysed several physicochemical properties such as the aliphatic index, Boman index, hydrophobic ratio, instability index, isoelectric point, molecular weight, sequence charge, and sequence length, which were all calculated using ModlAMP v.4.3.0. [74]. As physicochemical features represent non-parametric and dependent data, significant differences were considered if *p* < 0.05 by using the Friedman tests with truncated data, followed by Wilcoxon Signed-Rank tests with Bonferroni corrections for pairwise comparisons [75,76]. The final nr dataset of the non-toxic and non-haemolytic AMPs was used for further analysis.

### 4.5. Selection of Cnidaria Singular AMPs (CnSA) Using Complex Network Analyses

To identify unique AMPs from Cnidaria, we compared our non-redundant(nr), non-haemolytic, and non-toxic composite dataset against two public AMP databases—StarPepDB [9] and DRAMP [10], using CD-HIT-2D v4.8.1 [66] at 0.40 identity cutoff. Before comparison, we pre-processed the StarPepDB and DRAMP datasets by applying CD-HIT v4.8.1 [66] at 0.98 sequence identity and retaining AMPs between 10 and 100 AA. Cnidarian AMPs were considered singular if their sequence identity with StarPepDB and DRAMP members was below 0.40. Otherwise, they were considered related. We employed SeqDivA v1.0 [77] and Dover Analyser v.0.1.2 [78] to generate heatmaps and histograms depicting the sequence identity landscape among the Cnidaria Singular AMPs (CnSA). Then, the sequence spaces of StarPepDB, DRAMP, and the non-redundant, non-haemolytic, and non-toxic CnSAs were projected within a half-space proximity network (HSPN) [79], constructed using StarPep Toolbox [80]. The HSPN illustrates the topological relationship between the three previously mentioned datasets, where each node/peptide was represented by an optimal set of molecular descriptors. Pairwise alignment-free (AF) similarity associations were determined using the Euclidean distance metric with min–max normalization [36]. Then, the modulatory optimization algorithm based on the Louvain method was applied to cluster the AMPs within the HSPN [81]. Each cluster of the CnSAs was characterized physiochemically using ModlAMP v.4.3.0. [74] by estimating the aliphatic index, Boman index, hydrophobic ratio, isoelectric point, sequence charge, and sequence length. The mean value for each property was calculated.

To further extract the most representative AMPs from the CnSA dataset, the HSPN was exclusively built up with CnSA (Cnidarian AMPs with sequence identity <0.40 with StarPepDB and DRAMP chemical space). Then, clusters/communities in the HSPN using the Louvain method [81] were identified, and afterwards, two centrality measures, harmonic (HC) [82] and hub-bridge (HB) centrality [83], were calculated. The AMPs were ranked down according to their centrality values and reduced by applying local alignment comparison at 0.35 of sequence identity (node tables with rankings are available in Supplementary Table S5). Thus, two representative datasets were extracted: the union and intersection of the peptides identified by HC and HB centralities. Both datasets, CnSA and the representative CnSA (extracted from the intersection of HC and HB centralities), were projected into the HSPN and built up with StarPepDB and DRAMP.

### 4.6. Activity and Strain-Specific Predictions

To identify promising peptides with antibacterial (ABP), antiviral (AVP), antifungal (AFP), and anticancer (ACP) activities, we screened the representative CnSA dataset derived from the union dataset of the HC and HB centralities. Predictions were made using two main tools: the standalone version of iAMPCN [84], a DL framework based on convolutional neural networks, and the AMPDiscover [85] web server, an RF-based classifier. ABPs, AVPs, and AFPs were predicted using both tools, while ACPs were exclusively predicted by iAMPCN. To enhance robustness, we incorporated consensus predictions for each activity using complementary models: (I) ABPs: Predictions were refined with the AntiBP3 [86] web interface based on SVM classifier (Gram-variable ABP model, threshold = 0.51); (II) AVPs: Predictions were cross-validated with AI4AVP [87], a DL tool using the PC6 protein-encoding method; (III) AFPs: Cross-checked with Antifp [88] web server, an SVM-based method (AntifpMain_binary_model3, threshold = 0.5); (IV) ACPs: Additional predictions used AntiCP 2.0 [89] webserver, SVM-based (model2, threshold = 0.5), and ModlAMP v4.3.0. [74] (SVM model). ACPs shorter than 38 AA were further filtered, and we used AcPEP [90] to identify potential targets via DL methods based on convolutional neural network and multitask learning. We then generated Venn diagrams to explore the union and intersection datasets for each peptide type (ABP, AVP, AFP, and ACP). For further refinement, strain-specific activity predictions were performed for ABPs, AFPs, and AVPs using DBAASP [11]. Specifically, for ABPs, three strain-specific antibacterial prediction models were employed [91,92]: Model I: ML predictions based on AMP sequence data; Model II: Cluster-based predictions using peptide sequence data; and Model III: ML predictions integrating peptide and bacterial genome data. These were used to rank the ABPs against some important ESKAPEE pathogens [57] but also *Bacillus subtilis*. The predicted ABPs were hierarchically ranked according to the following: Rank I—ABPs intersecting all three antibacterial activity models and all three strain-specific models; Rank II—ABPs intersecting all three activity models and two strain-specific models; Rank III—ABPs in the union of activity models but intersecting all three strain-specific models (effective against 4–5 pathogens); and Rank IV—ABPs in the union of activity models intersecting all three strain-specific models.

## Figures and Tables

**Table 2:** Peptidome filtering results for each proteolysis protocol. The number of putative antimicrobial proteins (AMP precursors) in each database after AMPir prediction (precursor model) is shown in brackets. Each protein library was subjected to a one-enzyme proteolysis protocol using five different proteases—AspN/aspn, Chymotrypsin/chym, GluC/gluc, Proteinase K/protk, and Trypsin/tryp. The number of peptides decreased with each filtering step, resulting in non-redundant (nr) peptidomes with AMP-like characteristics for each database and protease combination.

Proteolysis Protocol	Total Peptides	6–40 AA Length	Non-Duplicated Peptides	Non-Redundant Peptides	20 AA Alphabet
db1_aspn	15,851	8432	7980	4376	4376
db1_chym	32,418	9242	8565	2696	2696
db1_gluc	15,240	8172	7649	4447	4447
db1_protk	68,118	3285	2999	175	175
db1_tryp	19,198	9170	8442	4318	4318
db2_aspn	2748	1422	1311	698	698
db2_chym	5075	1479	1338	434	434
db2_gluc	2210	1261	1158	744	744
db2_protk	10,692	555	494	25	25
db2_tryp	3122	1376	1257	670	670
db3_aspn	73,527,51	4,175,173	3,694,840	2,115,242	1,820,209
db3_chym	18,999,471	3,996,393	3,311,893	858,127	818,622
db3_gluc	5,438,132	3,348,195	3,021,505	1,947,320	1,609,672
db3_protk	33,464,859	1,417,423	1,040,671	59,408	58,164
db3_tryp	8,467,153	4,350,869	3,846,043	2,082,476	1,849,069
db4_aspn	2,291,447	1,292,563	1,205,239	682,256	586,367
db4_chym	6,080,475	1,207,735	1,090,192	262,041	250,336
db4_gluc	1,702,547	1,044,291	982,746	629,228	519,668
db4_protk	10,512,274	417,458	357,444	17,402	17,080
db4_tryp	2,630,768	1,343,898	1,252,674	673,179	595,910
db5_aspn	4,031,801	2,293,354	2,038,733	1,169,051	996,612
db5_chym	10,705,538	2,166,093	1,826,084	457,572	436,127
db5_gluc	2,983,890	1,843,785	1,666,856	1,080,419	883,537
db5_protk	18,562,675	763,345	591,295	31,353	30,722
db5_tryp	4,665,535	2,393,038	2,126,340	1,151,478	1,014,421
db6_aspn	670,520	381,979	370,092	208,598	177,282
db6_chym	1,831,070	348,971	333,615	72,869	69,353
db6_gluc	482,489	300,680	292,545	190,074	153,954
db6_protk	3,089,109	119,570	112,026	4804	4704
db6_tryp	768,090	397,489	384,873	204,191	179,035
db7_aspn	380,990	215,028	200,493	113,076	97,943
db7_chym	932,823	210,478	193,912	52,098	49,536
db7_gluc	279,267	171,878	161,719	104,066	86,699
db7_protk	1,682,862	78,598	70,910	3998	3909
db7_tryp	424,506	222,032	207,692	112,808	100,524
				Total Peptides	12,428,038
				Total nr Peptides	8,278,560

**Table 3:** Summary of AMP mining results following in silico proteolysis. The table demonstrates the progression of the 35 initial datasets into robust, consensus detections of three peptide types: (**1**) AMPs, (**2**) Non-Haemolytic AMPs, and (**3**) Non-Haemolytic and Non-Toxic AMPs. This was achieved using three prediction tools at each mining step (Supplementary Figures S2–S4). The total number of peptides before and after removal of non-redundant (nr) peptides identified via CD-HIT at a 0.98 similarity threshold are represented.

Proteolysis Protocol	Peptidomes	(1) AMP	(2) Non-Haemolytic AMP	(3) Non-Haemolytic and Non-Toxic AMP
db1_aspn	4376	214	36	9
db1_chym	2696	35	9	3
db1_gluc	4447	298	82	20
db1_protk	175	0	0	0
db1_tryp	4318	49	8	4
Peptides db1	16,012	596	135	36
nr Peptides db1	10,955	537	133	36
db2_aspn	698	31	4	0
db2_chym	434	7	2	0
db2_gluc	744	47	15	1
db2_protk	25	1	0	0
db2_tryp	670	11	3	1
Peptides db2	2571	97	24	2
nr Peptides db2	1788	90	24	2
db3_aspn	1,820,209	134,007	21,138	5055
db3_chym	818,622	14,534	3665	946
db3_gluc	1,609,672	78,250	26,331	4974
db3_protk	58,164	456	96	9
db3_tryp	1,849,069	38,029	12,366	4023
Peptides db3	6,155,736	265,276	63,596	15,007
nr Peptides db3	4,229,977	244,894	62,423	14,816
db4_aspn	586,367	42,756	6531	1486
db4_chym	250,336	4260	1033	269
db4_gluc	519,668	24,700	8248	1586
db4_protk	17,080	111	22	3
db4_tryp	595,910	12,479	4080	1356
Peptides db4	1,969,361	84,306	19,950	4700
nr Peptides db4	1,357,350	77,857	19,583	4638
db5_aspn	996,612	72,434	11,325	2633
db5_chym	436,127	3929	934	234
db5_gluc	883,537	40,721	13,585	2640
db5_protk	30,722	239	56	11
db5_tryp	1,014,421	20,694	6565	2134
Peptides db5	3,361,419	138,017	32,465	7652
nr Peptides db5	2,316,742	127,952	31,909	7580
db6_aspn	177,282	12,861	1907	389
db6_chym	69,353	1162	292	70
db6_gluc	153,954	6591	2300	416
db6_protk	4704	21	2	0
db6_tryp	179,035	3800	1204	375
Peptides db6	584,328	24,435	5705	1250
nr Peptides db6	404,314	22,683	5585	1237
db7_aspn	97,943	6914	1138	299
db7_chym	49,536	937	254	54
db7_gluc	86,699	4444	1486	320
db7_protk	3909	45	7	0
db7_tryp	100,524	2029	643	208
Peptides db7	338,611	14,369	3528	881
nr Peptides db7	230,192	13,177	3456	867
Total Peptides	12,428,038	527,096	125,403	29,528
Total nr Peptides	8,278,560	473,747	119,531	28,279

**Table 4:** Summary of the 20 candidate ABPs (antibacterial peptides) obtained from omics data of Cnidaria identified through all the mining steps. These candidates are ranked based on prediction strength and model consensus: Rank I (ABPs intersecting all three antibacterial activity models and all three strain-specific models); Rank II (ABPs intersecting all three activity models and two strain-specific models); and Rank III (ABPs in the union of activity models but intersecting all three strain-specific models (effective against 4–5 pathogens)). These peptides are recommended for further validation through in vitro testing.

Species	Subphylum	Class (Order)	Candidate ABP Sequence	Rank	Predicted Antimicrobial Activity
*Ctenactis echinata*	Anth.	Hexacorallia (Scleractinia)	CGVWQYRQGNSLYVQVISRPKKSGFRFR	I	*B. subtilis*
*Galaxea fascicularis*	Anth.	Hexacorallia (Scleractinia)	DLFFRFVNYLGNQYNQLGWWKKVRSSGSRG	I	*B. subtilis*
*Favites colemani*	Anth.	Hexacorallia (Scleractinia)	DRFGKEEKQWPFVPWQWPVRRNVLLRRQR	I	*B. subtilis*
Catalaphyllia jardinei *	Anth.	Hexacorallia (Scleractinia)	GAWSGAKRYGTGQRHISSNSSLFRKWGND	I	*B. subtilis*
*Fimbriaphyllia ancora*	Anth.	Hexacorallia (Scleractinia)	VFPRFRSIFSPGVTRGLRAVSSLSKD	I	*B. subtilis*, *E. coli*, *P. aeruginosa*
*Alveopora japonica*	Anth.	Hexacorallia (Scleractinia)	CRKQVYKPPLQFSGLSSSSFLSYLVKRFNTQQRGSFWR	II	*B. subtilis*, *K. pneumoniae*
*Heliopora coerulea*	Anth.	Octocorallia (Scleralcyonacea)	PMKAWITGIAANRGTKGGSAKCAVGLFKSRVKD	II	*E. coli*
*Protopalythoa variabilis*	Anth.	Hexacorallia (Zoantharia)	QPRLIFFGSTSSFRAPHGQQKQVHKFAAKVQCCK	II	*E. coli*
*Acropora millepora*	Anth.	Hexacorallia (Scleractinia)	RGQWQINKRTGSKSCARLKTTGAPHMASGWQVWK	II	*B. subtilis*, *E. coli*
Goniopora lobata *	Anth.	Hexacorallia (Scleractinia)	RGRKLCLPWTFWLGSRTVIQGRCTQPASASGSKGPQRRF	II	*B. subtilis*, *E. coli*, *K. pneumoniae*, *P. aeruginosa*, *S. aureus*
Chironex fleckeri *	Med.	Cubozoa (Chirodropida)	RWRNVNGWGKSKKKNANGSHIGLWLTGGGG	II	*B. subtilis*, *E. coli*, *K. pneumoniae*
*Fimbriaphyllia ancora*	Anth.	Hexacorallia (Scleractinia)	TLNIPVAGGTKSTAGMWRRCWNGAVPSRTPSKRFG	II	*B. subtilis*, *E. coli*, *P. aeruginosa*
*Alveopora japonica*	Anth.	Hexacorallia (Scleractinia)	YYWNPRLRPGLQVSCSHGSCKTSLAFGRLLKSKD	II	*B. subtilis*
*Ricordea yuma*	Anth.	Hexacorallia (Corallimorpharia)	CRSNRTQQWGLGSYIRILGRASVVTLKQPL	III	*B. subtilis*, *E. coli*, *K. pneumoniae*, *P. aeruginosa*
*Montipora digitata*	Anth.	Hexacorallia (Scleractinia)	CSMRPISSSWLRFSKKIWSTSAR	III	*B. subtilis*, *E. coli*, *K. pneumoniae*, *P. aeruginosa*
Phyllodiscus semoni *	Anth.	Hexacorallia (Actiniaria)	CWTWVATPTFAHGMVQVWRASQRVRSRLTN	III	*B. subtilis*, *E. coli*, *P. aeruginosa*, *S. aureus*
*Polymyces wellsi*	Anth.	Hexacorallia (Scleractinia)	NISFNSSASGRSLFGHFGRFRTLSWLRGWGG	III	*B. subtilis*, *E. coli*, *K. pneumoniae*, *P. aeruginosa*, *S. aureus*
*Goniopora norfolkensis*	Anth.	Hexacorallia (Scleractinia)	RPAISGAVTISGKFQKAWGSVHKPLNRCRSSLWGGG	III	*B. subtilis*, *E. coli*, *K. pneumoniae*, *P. aeruginosa*, *S. aureus*
*Goniopora norfolkensis*	Anth.	Hexacorallia (Scleractinia)	SGLRKSRMMKWPLSTGGRWSRGGLVA	III	*E. coli*, *K. pneumoniae*, *P. aeruginosa*, *S. aureus*
*Galaxea fascicularis*	Anth.	Hexacorallia (Scleractinia)	YPKPSLANWTRSSGTSIKGKLWLTGRHPHLRAGSG	III	*E. coli*, *K. pneumoniae*, *P. aeruginosa*, *S. aureus*

Anth.—Anthozoa; Med.—Medusozoa; Strains—*Bacillus subtilis*, *Escherichia coli*, *Klebsiella pneumoniae*, *Pseudomonas aeruginosa* and *Staphylococcus aureus*. * Samples from tentacles.

## Data Availability

The peptide datasets generated in this study are publicly available through the Mendeley Data Repository. The datasets are listed in the following order, corresponding to their citation order in the text: Dataset 1. Protein Libraries Of Seven Databases From Cnidaria Omics Data After Duplicates Removal (Mendeley Data, V1, doi: 10.17632/grwy638mtr.1). Dataset 2. Protein Libraries Of Seven Databases From Cnidaria Omics Data After Duplicates Removal and AMPir Prediction (Mendeley Data, V1, doi: 10.17632/myp4j56gpz.1). Dataset 3. Generated Peptide Libraries From Cnidaria Omics Data After Applying The In Silico Enzymatic Digestion Protocols (Mendeley Data, V1, doi: 10.17632/mx4fkk4v8j.1). Dataset 4. Outputs From The Antimicrobial, Haemolytic, And Toxicity Prediction Tools From Cnidaria Omics Data (Mendeley Data, V1, doi: 10.17632/xhxjf6tzv9.1). Dataset 5. Consensus Antimicrobial Peptides Identified By Three Prediction Models (AMPir, AMPlify and Macrel) From Peptidomes Derived From Cnidaria Omics Data (Mendeley Data, V1, doi: 10.17632/vn5mk4d44m.1). Dataset 6. Consensus Non-Haemolytic Antimicrobial Peptides Identified By Three Prediction Models (HemoPi, Macrel and MQSSM) From Peptidomes Derived From Cnidaria Omics Data (Mendeley Data, V1, doi: 10.17632/dc5c6gb2w6.1). Dataset 7. Consensus Non-Haemolytic And Non-Toxic Antimicrobial Peptides Identified By Three Prediction Models (CAPTP, ToxinPred3, and ToxTeller) From Peptidomes Derived From Cnidaria Omics Data (Mendeley Data, V1, doi: 10.17632/fpb8nvhvh2.1). Dataset 8. Non-redundant DRAMP and StarPepDB Databases, Including Sequences With Less Than 0.40 Similarity To Both Databases And The Resulting Cnidaria Singular AMPs (CnSA) (Mendeley Data, V1, doi: 10.17632/jh6fcc69r9.1). Dataset 9. Representative Sets Of Singular Non-haemolytic, Non-Toxic AMPs From Cnidaria Extracted Via Harmonic And Hub-Bridge Network Centralities, Including The Union And Intersection Datasets (Mendeley Data, V1, doi: 10.17632/gdrn9sypx4.1). Dataset 10: Outputs Of The Activity And Strain Specific Predictions From Cnidaria Omics Data (Mendeley Data, V1, doi: 10.17632/rrmym7s4pc.1). Dataset 11: Union And Intersection Datasets Of The Predicted Antibacterial, Antiviral, Antifungal, And Anticancer Peptides From Cnidaria Omics Data (Mendeley Data, V1, doi: 10.17632/8vbphryhp4.1). Dataset 12. Candidate ABPs From Rank I, II, III, and IV Obtained From Cnidaria Omics Data (Mendeley Data, V1, doi: 10.17632/bw472ms3vt.1). All standalone software and web servers used for mining AMPs from omics data are freely available as indicated in the text. Specifically, our in-house (StarPep) software for complex network analyses and visualization is publicly available at https://github.com/Grupo-Medicina-Molecular-y-Traslacional/StarPep, (accessed on 19 August 2024) and the online documentation is available at https://grupo-medicina-molecular-y-traslacional.github.io/StarPep_doc (accessed on 19 August 2024).

## Supplementary Materials

The following supporting information can be downloaded at: https://www.mdpi.com/article/10.3390/molecules30030550/s1, Figure S1: Completeness scores of the 27 assembled transcriptomes obtained from SRA data (NCBI), assessed with BUSCO using the Metazoa dataset; Figure S2: Venn diagrams representing the number of predicted AMPs by AMPir, AMPlify, and Macrel from each protease–database combination; Figure S3: Venn diagrams representing the number of predicted non-haemolytic AMPs by HemoPi, Macrel, and MQSSM from each protease–database pair; Figure S4: Venn diagrams representing the number of predicted non-haemolytic and non-toxic AMPs by CAPTP, ToxinPred3, and ToxTeller from each protease–database pair; Figure S5: HSPN clustering of the 1017 AMPs obtained from the intersection of the Harmonic and Hub-Bridge datasets within the Cnidaria Singular AMPs (CnSA); Figure S6: Strain-specific activity predictions for ABPs derived from the most representative CnSA datasets and Venn diagrams summarizing activity predictions for five bacterial strains—*Bacillus subtilis*, *Escherichia coli*, Klebsiella pneumoniae, *Pseudomonas aeruginosa,* and *Staphylococcus aureus*. Figure S7: Distribution of the 152 predicted antimicrobial peptides (ABPs) across various categories; Figure S8: Antifungal and antiviral specific predictions for both intersection and union datasets in DBAASP; Table S1: Overview of Cnidarian omics data used in this study; Table S2: BUSCO statistics for the 27 SRA-derived transcriptomes; Table S3: Number of proteins in each database, provided in three stages: original dataset, after duplicate removal and following AMPir prediction filtering, with statistics included; Table S4: Statistics for each mining step (peptides, AMPs, and non-haemolytic and non-toxic AMPs); Table S5: Characterization of the CnSA within the HSPN, detailing properties for each of the 3310 nodes, and the HC and HB values; Table S6: Overview of the 152 predicted ABPs obtained from omics data of Cnidaria.
