# Supplementary material for: Unlocking Antimicrobial Peptides: In Silico Proteolysis and Artificial Intelligence-Driven Discovery from Cnidarian Omics
Source: Molecules. 2025 Jan 25;30(3):550. doi: 10.3390/molecules30030550 (PMC11820242; doi:10.3390/molecules30030550)
Supplement: Supplementary file 1 [file molecules-30-00550-s001.zip › Supplementary_Information_Vfinal.pdf]

Supplementary Material

Unlocking Antimicrobial Peptides: In Silico Proteolysis and Artificial Intelligence-Driven Discovery from Cnidarian Omics

Ricardo Alexandre Barroso [1,2], Guillermin Agüero-Chapin [1,2], Rita Sousa [1,2], Yovani Marrero-Ponce [3,4] and Agostinho Antunes [1,2,*]

Table S1: Overview of Cnidarian omics data used in this study

Table S2: BUSCO statistics for the 27 SRA-derived Transcriptomes

Table S3: Number of proteins in each database, provided in three stages: original dataset, after duplicate removal and following AMPir prediction filtering, with statistics included

Table S4: Statistics for each mining step (peptides, AMPs, and non-haemolytic and non-toxic AMPs)

Table S5 (Excel file): Characterization of the CnSA within the HSPN, detailing properties for each of the 3310 nodes, and the HC and HB values

Table S6 (Excel file): Overview of the 152 predicted ABPs obtained from omics data of Cnidaria.

Figure S1: Completeness scores of the 27 assembled transcriptomes obtained from SRA data (NCBI), assessed with BUSCO using the Metazoa dataset

Figure S2: Venn diagrams representing the number of predicted AMPs by AMPir, AMPlify, and Macrel from each protease–database combination

Figure S3: Venn diagrams representing the number of predicted non-haemolytic AMPs by HemoPi, Macrel, and MQSSM from each protease–database pair

Figure S4: Venn diagrams representing the number of predicted non-haemolytic and non-toxic AMPs by CAPTP, ToxinPred3, and ToxTeller from each protease–database pair

Figure S5: HSPN clustering of the 1017 AMPs obtained from the intersection of the Harmonic and Hub-Bridge datasets within the Cnidaria Singular AMPs (CnSA)

Figure S6: Strain-specific activity predictions for ABPs derived from the most representative CnSA datasets and Venn diagrams summarizing activity predictions for five bacterial strains—*Bacillus subtilis, Escherichia coli, Klebsiella pneumoniae, Pseudomonas aeruginosa,* and *Staphylococcus aureus*

Figure S7: Distribution of the 152 predicted antimicrobial peptides (ABPs) across various categories

Figure S8: Antifungal and antiviral specific predictions for both intersection and union datasets in DBAASP

Table S1. Overview of Cnidarian omics data used in this study, including details on database sources, tissue types, and protein counts.

Species	Database	Subphylum	Group	UniProt/TSA/SRA Code	Date	Info	Protein Count
Actinia tenebrosa	1	Anthozoa	Hexacorallia, Actiniaria	UP000515163/GCF_009602425.1	06/11/2019	Non-specific / Whole Body	23842
Nematostella vectensis	1	Anthozoa	Hexacorallia, Actiniaria	UP000001593/GCA_000209225.1	22/08/2007	Non-specific / Whole Body	24435
Desmophyllum pertusum	1	Anthozoa	Hexacorallia, Scleractinia	UP001163046/GCA_029204205.1	17/04/2019	Non-specific / Whole Body	37379
Pocillopora damicornis	1	Anthozoa	Hexacorallia, Scleractinia	UP000275408/GCA_003704095.1	31/10/2018	Non-specific / Whole Body	25411
Stylophora pistillata	1	Anthozoa	Hexacorallia, Scleractinia	UP000225706/GCA_002571385.2	17/10/2017	Non-specific / Whole Body	24067
Paramuricea clavata	1	Anthozoa	Octocorallia, Malacalcyonacea	UP001152795/GCA_902702795.2	08/11/2022	Non-specific / Whole Body	69559
Clytia hemisphaerica	2	Medusozoa	Hydrozoa	UP000594262/GCA_902728285.1	04/02/2020	Non-specific / Whole Body	24557
Hydra vulgaris	2	Medusozoa	Hydrozoa	UP000694840/GCF_000004095.1	30/10/2009	Non-specific / Whole Body	21303
Acanthogorgia aspera	3	Anthozoa	Octocorallia, Malacalcyonacea	GEXC00000000.1	14/09/2016	Non-specific / Whole Body	81723
Acropora cerviconis	3	Anthozoa	Hexacorallia, Scleractinia	GJIG00000000.1	30/08/2022	Non-specific / Whole Body	1267328
Acropora digitifera	3	Anthozoa	Hexacorallia, Scleractinia	GIVI00000000.1	13/11/2020	Non-specific / Whole Body	748853
Acropora gemmifera	3	Anthozoa	Hexacorallia, Scleractinia	GFQE00000000.1	03/10/2017	Non-specific / Whole Body	268146
Acropora hyancinthus	3	Anthozoa	Hexacorallia, Scleractinia	GDIF00000000.1	29/07/2015	Non-specific / Whole Body	66872
Acropora millepora	3	Anthozoa	Hexacorallia, Scleractinia	GHGU00000000.1	02/04/2019	Non-specific / Whole Body	721367
Acropora solitaryensis	3	Anthozoa	Hexacorallia, Scleractinia	ICPH00000000.1	27/09/2019	Non-specific / Whole Body	121395
Acropora tenuis	3	Anthozoa	Hexacorallia, Scleractinia	IADL00000000.1	28/08/2018	Non-specific / Whole Body	365224
Agaricia lamarcki	3	Anthozoa	Hexacorallia, Scleractinia	GGLC00000000.3	28/08/2018	Non-specific / Whole Body	435762
Alveopora japonica	3	Anthozoa	Hexacorallia, Scleractinia	GGJR00000000.1	19/03/2018	Non-specific / Whole Body	961641
Anthopleura elegantissima	3	Anthozoa	Hexacorallia, Actiniaria	GBXJ00000000.1	20/11/2014	Non-specific / Whole Body	787875
Astroides calycularis	3	Anthozoa	Hexacorallia, Scleractinia	GIRZ00000000.1	23/07/2020	Non-specific / Whole Body	561718
Briareum asbestinum	3	Anthozoa	Octocorallia, Scleralcyonacea	GHBD00000000.2	15/08/2019	Non-specific / Whole Body	613438
Calyptrophora lyra	3	Anthozoa	Octocorallia, Scleralcyonacea	GJII00000000.1	17/03/2022	Non-specific / Whole Body	470134
Chrysogorgia stellata	3	Anthozoa	Octocorallia, Scleralcyonacea	GJIJ00000000.1	17/03/2022	Non-specific / Whole Body	569607
Clavularia sp.	3	Anthozoa	Octocorallia, Malacalcyonacea	GHAW00000000.1	14/11/2018	Non-specific / Whole Body	614091
Corynactis australis	3	Anthozoa	Hexacorallia, Corallimorpharia	GELM00000000.1	01/11/2016	Non-specific / Whole Body	167069
Ctenactis echinata	3	Anthozoa	Hexacorallia, Scleractinia	GDZV00000000.1	10/11/2015	Non-specific / Whole Body	87019
Cyphastrea serailia	3	Anthozoa	Hexacorallia, Scleractinia	GETH00000000.1	16/06/2016	Non-specific / Whole Body	193524
Edwardsiella carnea	3	Anthozoa	Hexacorallia, Actiniaria	GGGD00000000.1	14/07/2018	Non-specific / Whole Body	475457
Eleutherobia rubra	3	Anthozoa	Octocorallia, Malacalcyonacea	GHFI00000000.1	12/02/2019	Non-specific / Whole Body	325194
Favia/Dipsastraea lizadensis	3	Anthozoa	Hexacorallia, Scleractinia	GDZU00000000.1	10/11/2015	Non-specific / Whole Body	110215
Favites colemani	3	Anthozoa	Hexacorallia, Scleractinia	GIVN00000000.1	13/11/2020	Non-specific / Whole Body	735877
Fimbriaphyllia ancora	3	Anthozoa	Hexacorallia, Scleractinia	ICQS00000000.1	16/09/2020	Non-specific / Whole Body	1082203
Galaxea fascicularis	3	Anthozoa	Hexacorallia, Scleractinia	GFAZ00000000.1	17/04/2017	Non-specific / Whole Body	1010746
Heliopora coerulea	3	Anthozoa	Octocorallia, Scleralcyonacea	GFVH00000000.1	07/03/2018	Non-specific / Whole Body	299914

Metridium senile	3	Anthozoa	Hexacorallia, Actiniaria	GGGC00000000.1	27/02/2019	Non-specific / Whole Body	127132
Montipora capitata	3	Anthozoa	Hexacorallia, Scleractinia	GFRO00000000.1	17/07/2017	Non-specific / Whole Body	104929
Montipora digitata	3	Anthozoa	Hexacorallia, Scleractinia	GIVM00000000.1	13/11/2020	Non-specific / Whole Body	606544
Palythoa caribaeorum	3	Anthozoa	Hexacorallia, Zoantharia	GESO00000000.1	01/06/2016	Non-specific / Whole Body	459582
Palythoa sp.	3	Anthozoa	Hexacorallia, Zoantharia	GGUI00000000.1	09/10/2019	Non-specific / Whole Body	561450
Pocillopora acuta	3	Anthozoa	Hexacorallia, Scleractinia	GGRP00000000.1	10/07/2019	Non-specific / Whole Body	89222
Pocillopora verrucosa	3	Anthozoa	Hexacorallia, Scleractinia	GJZD00000000.1	10/01/2023	Non-specific / Whole Body	364512
Polymyces wellsi	3	Anthozoa	Hexacorallia, Scleractinia	GKDK00000000.1	10/07/2023	Non-specific / Whole Body	411660
Porites lutea	3	Anthozoa	Hexacorallia, Scleractinia	GGER00000000.1	19/01/2018	Non-specific / Whole Body	416366
Protopalythoa variabilis	3	Anthozoa	Hexacorallia, Zoantharia	GCVI00000000.1	11/04/2016	Non-specific / Whole Body	407283
Renilla muelleri	3	Anthozoa	Octocorallia, Scleralcyonacea	GJZZ00000000.1	02/08/2022	Non-specific / Whole Body	119186
Rhodactis indosinensis	3	Anthozoa	Hexacorallia, Corallimorpharia	GELO00000000.1	01/11/2016	Non-specific / Whole Body	169678
Rhodaniridogorgia sp.	3	Anthozoa	Octocorallia, Scleralcyonacea	GJIH00000000.1	17/03/2022	Non-specific / Whole Body	600342
Ricordea yuma	3	Anthozoa	Hexacorallia, Corallimorpharia	GELN00000000.1	01/11/2016	Non-specific / Whole Body	128218
Scleronephthya gracillima	3	Anthozoa	Octocorallia, Malacalcyonacea	GJHC00000000.1	28/07/2021	Non-specific / Whole Body	69225
Scolanthus callimorphus	3	Anthozoa	Hexacorallia, Actiniaria	GGGE00000000.1	27/02/2019	Non-specific / Whole Body	622171
Seriatropora caliendrum	3	Anthozoa	Hexacorallia, Scleractinia	GIVG00000000.1	13/11/2020	Non-specific / Whole Body	682598
Stichodactyla helianthus	3	Anthozoa	Hexacorallia, Actiniaria	GGNY00000000.1	22/05/2018	Non-specific / Whole Body	442313
Xenia sp.	3	Anthozoa	Octocorallia, Malacalcyonacea	GHBC00000000.1	28/01/2019	Non-specific / Whole Body	438621
Zoanthus sp.	3	Anthozoa	Hexacorallia, Zoantharia	GGTW00000000.1	07/09/2018	Non-specific / Whole Body	590885
Alatina alata	4	Medusozoa	Cubozoa	GEUJ00000000.1	05/07/2016	Non-specific / Whole Body	110225
Calvadosia cruxmelitensis	4	Medusozoa	Staurozoa	HAHC00000000.1	29/09/2017	Non-specific / Whole Body	286864
Cassiopea andromeda	4	Medusozoa	Scyphozoa	GJJJ00000000.1	03/10/2022	Non-specific / Whole Body	149941
Chrysaora quinquecirrha	4	Medusozoa	Scyphozoa	GILX00000000.1	29/04/2020	Non-specific / Whole Body	338074
Copula sivickisi	4	Medusozoa	Cubozoa	GHBG00000000.1	28/01/2019	Non-specific / Whole Body	417516
Craterolophus convolvulus	4	Medusozoa	Staurozoa	HAGZ00000000.1	29/09/2017	Non-specific / Whole Body	140294
Dynamena pumila	4	Medusozoa	Hydrozoa	GHMC00000000.1	29/05/2019	Non-specific / Whole Body	193769
Haliclystus auricula	4	Medusozoa	Staurozoa	HAHA00000000.1	29/09/2017	Non-specific / Whole Body	128662
Haliclystus sanjuanensis	4	Medusozoa	Staurozoa	HAHB00000000.1	29/09/2017	Non-specific / Whole Body	157217
Hydra oligactis	4	Medusozoa	Hydrozoa	GHUC00000000.1	08/09/2020	Non-specific / Whole Body	70952
Hydractinia symbiolongicarpus	4	Medusozoa	Hydrozoa	GJUZ00000000.1	17/03/2022	Non-specific / Whole Body	222167
Lucernaria quadricornis	4	Medusozoa	Staurozoa	HAHD00000000.1	29/09/2017	Non-specific / Whole Body	300790
Millepora alcicornis	4	Medusozoa	Hydrozoa	GKBI00000000.1	09/09/2022	Non-specific / Whole Body	138972
Millepora complanata	4	Medusozoa	Hydrozoa	GIXI00000000.1	30/08/2022	Non-specific / Whole Body	673826
Millepora squarrosa	4	Medusozoa	Hydrozoa	GFGU00000000.1	07/03/2018	Non-specific / Whole Body	797586
Morbakka virulenta	4	Medusozoa	Cubozoa	GHAF00000000.1	08/11/2018	Non-specific / Whole Body	197384
Phialella quadrata	4	Medusozoa	Hydrozoa	GKAK00000000.1	09/01/2023	Non-specific / Whole Body	64221

Physalia physalis	4	Medusozoa	Hydrozoa	GHBB00000000.1	28/01/2019	Non-specific / Whole Body	74421
Podocoryna carnea	4	Medusozoa	Hydrozoa	GJNL00000000.1	21/11/2022	Non-specific / Whole Body	811493
Porpita porpita	4	Medusozoa	Hydrozoa	GHBA00000000.1	28/01/2019	Non-specific / Whole Body	146355
Rathkea octopunctata	4	Medusozoa	Hydrozoa	GKAO00000000.1	09/01/2023	Non-specific / Whole Body	56744
Rhopilema esculentum	4	Medusozoa	Scyphozoa	GGZZ00000000.1	31/10/2019	Non-specific / Whole Body	185257
Turritopsis sp.	4	Medusozoa	Hydrozoa	IAAF00000000.1	17/08/2016	Non-specific / Whole Body	240888
Velella velella	4	Medusozoa	Hydrozoa	GHAZ00000000.1	28/01/2019	Non-specific / Whole Body	441513
Anemonia viridis	5	Anthozoa	Hexacorallia, Actiniaria	SRR1573633	11/09/2015	Tentacles	258361
Catalaphyllia jardinei	5	Anthozoa	Hexacorallia, Scleractinia	SRR18847906	21/04/2022	Tentacles	402397
Ceriantheomorphe brasiliensis	5	Anthozoa	Hexacorallia, Ceriantharia	SRR11802642	25/07/2020	Tentacles	214753
Dofleinia cf. armata	5	Anthozoa	Hexacorallia, Actiniaria	ERR6139597	10/11/2021	Tentacles	233127
Entacmaea quadricolor	5	Anthozoa	Hexacorallia, Actiniaria	GJFF00000000.1	19/04/2022	Tentacles	475573
Exaiptasia diaphana	5	Anthozoa	Hexacorallia, Actiniaria	SRR24433030	26/09/2023	Tentacles	1258938
Goniopora lobata	5	Anthozoa	Hexacorallia, Scleractinia	SRR18458550	24/03/2022	Tentacles	818587
Goniopora norfolkensis	5	Anthozoa	Hexacorallia, Scleractinia	SRR18458548	24/03/2022	Tentacles	943477
Goniopora planulata	5	Anthozoa	Hexacorallia, Scleractinia	SRR18458549	24/03/2022	Tentacles	931156
Haliplanella luciae	5	Anthozoa	Hexacorallia, Actiniaria	SRR7495168	13/11/2018	Tentacles	148450
Heteractis aurora	5	Anthozoa	Hexacorallia, Actiniaria	GJFG00000000.1	19/04/2022	Tentacles	458571
Heteractis crispa	5	Anthozoa	Hexacorallia, Actiniaria	GJFA00000000.1	18/04/2022	Tentacles	444825
Heteractis magnifica	5	Anthozoa	Hexacorallia, Actiniaria	GJFJ00000000.1	19/04/2022	Tentacles	488953
Heterodactyla hemprichii	5	Anthozoa	Hexacorallia, Actiniaria	SRR14115227	30/06/2021	Tentacles	503423
Isarachnanthus nocturnus	5	Anthozoa	Hexacorallia, Ceriantharia	SRR11802641	25/07/2020	Tentacles	199450
Macrodactyla doreensis	5	Anthozoa	Hexacorallia, Actiniaria	SRR14115222	30/06/2021	Tentacles	469869
Oulactis sp.	5	Anthozoa	Hexacorallia, Actiniaria	ERR2710216	03/03/2020	Tentacles	241076
Pachycerianthus borealis	5	Anthozoa	Hexacorallia, Ceriantharia	SRR11802643	25/07/2020	Tentacles	291992
Pachycerianthus maua	5	Anthozoa	Hexacorallia, Ceriantharia	SRR11802640	25/07/2020	Tentacles	273063
Phyllodiscus semoni	5	Anthozoa	Hexacorallia, Actiniaria	SRR14115230	30/06/2021	Tentacles	1255102
Renilla koellikeri	5	Anthozoa	Octocorallia, Scleralcyonacea	SRR7495167	13/11/2018	Tentacles	307772
Stichodactyla gigantea	5	Anthozoa	Hexacorallia, Actiniaria	GJFK00000000.1	19/04/2022	Tentacles	583020
Stichodactyla haddoni	5	Anthozoa	Hexacorallia, Actiniaria	GJFH00000000.1	05/04/2022	Tentacles	663175
Stichodactyla mertensii	5	Anthozoa	Hexacorallia, Actiniaria	GJFE00000000.1	19/04/2022	Tentacles	427025
Telmatactis stephensoni	5	Anthozoa	Hexacorallia, Actiniaria	SRR14511809	14/11/2021	Tentacles	137386
Aurelia aurita	6	Medusozoa	Scyphozoa	SRR8089702	23/10/2018	Tentacles	205131
Chironex fleckeri	6	Medusozoa	Cubozoa	SRR1819888	09/08/2015	Tentacles	189330
Chironex yamaguchii	6	Medusozoa	Cubozoa	SRR8101946	01/01/2019	Tentacles	261771
Chrysaora fuscescens	6	Medusozoa	Cubozoa	SRR3180892	27/02/2016	Tentacles	118738
Nemopilema nomurai	6	Medusozoa	Scyphozoa	SRR7754710	20/09/2019	Tentacles	476585
Sanderia malayensis	6	Medusozoa	Scyphozoa	SRR6298216	07/12/2018	Tentacles	124838
Tripedalia cystophora	6	Medusozoa	Cubozoa	SRR8101526	01/01/2019	Tentacles	278958
Cryptodendrum adhaesivum	7	Anthozoa	Hexacorallia, Actiniaria	SRR14115232	30/06/2021	Nematospheres	878716
Heterodactyla hemprichii	7	Anthozoa	Hexacorallia, Actiniaria	SRR14115226	30/06/2021	Nematospheres	313286

Table S2. BUSCO statistics for the 27 SRA-derived transcriptomes. C – Complete; S – Singular Copies; D – Duplicated Copies; F – Fragment Copies; M – Missing Copies.

Database	Species	Code	C	S	D	F	M
5	*Anemonia viridis*	SRR1573633	91,30%	54,80%	36,50%	6,00%	2,70%
5	*Dofleinia cf. Armata*	ERR6139597	93,90%	59,40%	34,50%	3,90%	2,20%
5	*Exaiptasia diaphana*	SRR24433030	98,80%	17,90%	80,90%	0,90%	0,30%
5	*Haliplanella luciae*	SRR7495168	76,90%	49,60%	27,30%	13,40%	9,70%
5	*Heterodactyla hemprichii*	SRR14115227	87,50%	65,20%	22,30%	9,10%	3,40%
5	*Macrodactyla doreensis*	SRR14115222	79,80%	53,00%	26,80%	11,80%	8,40%
5	*Oulactis sp.*	ERR2710216	94,30%	52,40%	41,90%	3,60%	2,10%
5	*Phyllodiscus semoni*	SRR14115230	98,20%	13,10%	85,10%	0,60%	1,20%
5	*Telmatactis stephensoni*	SRR14511809	55,50%	50,40%	5,10%	26,90%	17,60%
5	*Ceriantheomorphe brasiliensis*	SRR11802642	91,70%	66,00%	25,70%	4,80%	3,50%
5	*Isarachnanthus nocturnus*	SRR11802641	83,60%	55,70%	27,90%	9,40%	7,00%
5	*Pachycerianthus borealis*	SRR11802643	96,80%	50,90%	45,90%	1,30%	1,90%
5	*Pachycerianthus maua*	SRR11802640	82,40%	44,50%	37,90%	11,10%	6,50%
5	*Renilla koellikeri*	SRR7495167	96,30%	23,90%	72,40%	1,30%	2,40%
5	*Catalaphyllia jardinei*	SRR18847906	97,00%	46,10%	50,30%	1,80%	1,80%
5	*Goniopora lobata*	SRR18458550	96,60%	41,50%	55,50%	1,50%	1,50%
5	*Goniopora norfolkensis*	SRR18458548	96,60%	43,10%	53,60%	1,90%	1,50%
5	*Goniopora planulata*	SRR18458549	96,90%	44,70%	52,20%	1,90%	1,20%
6	*Chironex fleckeri*	SRR1819888	79,50%	68,30%	11,20%	10,50%	10,00%
6	*Chironex yamaguchii*	SRR8101946	89,40%	65,60%	23,80%	5,50%	5,10%
6	Chrysaora fuscescens	SRR3180892	58,30%	52,30%	6,00%	22,90%	18,80%
6	*Tripedalia cystophora*	SRR8101526	92,70%	71,80%	20,90%	4,00%	3,30%
6	*Aurelia aurita*	SRR8089702	73,80%	55,20%	18,60%	14,20%	12,00%
6	*Nemopilema nomurai*	SRR7754710	96,80%	26,60%	70,20%	1,30%	1,90%
6	*Sanderia malayensis*	SRR6298216	92,60%	72,20%	21,40%	3,20%	3,20%
7	*Cryptodendrum adhaesivum*	SRR14115232	90,10%	42,30%	47,80%	4,80%	5,10%
7	*Heterodactyla hemprichii*	SRR14115226	50,30%	43,50%	7,80%	31,00%	18,70%

Table S3. Number of proteins in each database, provided in three stages: original dataset, after duplicate removal (No_Dup) and following AMPir prediction filtering (No_Dup_AMPir). Statistics are included for each stage. No_Red_98% corresponds to protein counts after applying redundancy removal with CD-HIT at 0.98 sequence identity.

Database	Number of sequences	Sum of lengths	Minimum length	Average length	Maximum length
Original_1	204,693	87,052,731	8	425.3	22,539
Filtered_1_No_Dup	204,647	87,043,996	8	425.3	22,539
Filtered_1_No_Dup_AMPir	1473	147,689	45	100.3	627
Original_2	45,860	22,913,634	11	499.6	8,156
Filtered_2_No_Dup	45,859	22,913,498	11	499.7	8,156
Filtered_2_No_Dup_AMPir	191	23,419	55	112.6	360
Original_3	20,554,309	2,392,302,564	49	116.4	18,012
Filtered_3_No_Dup	15,491,809	1,917,236,786	49	123.8	18,012
Filtered_3_No_Dup_AMPir	1,045,220	70,691,396	49	67.6	606
Original_4	6,345,131	744,878,089	49	117.4	24,292
Filtered_4_No_Dup	5,018,612	617,800,624	49	123.1	24,292
Filtered_4_No_Dup_AMPir	326,045	21,851,044	49	67	607
Original_5	12,429,521	1,474,576,001	49	118.6	21,468
Filtered_5_No_Dup	8,338,288	1,079,249,660	49	129.4	21,468
Filtered_5_No_Dup_AMPir	579,611	38,945,446	49	67.2	609
Original_6	1,655,351	200,397,681	49	121.1	24,271
Filtered_6_No_Dup	1,132,735	149,146,332	49	131.7	24,271
Filtered_6_No_Dup_AMPir	96,921	6,412,716	49	66.2	586
Original_7	11,920,02	142,108,627	49	119.2	7,637
Filtered_7_No_Dup	925,924	117,808,010	49	127.2	7,637
Filtered_7_No_Dup_AMPir	53,236	3,632,718	49	68.2	305
NoAMPir_Composite_DB	31,157,874	3,991,198,906	8	128.1	24,292
NoAMPir_Composite_DB_No_Dup	30,626,016	3,942,328,738	8	128.7	24,292
NoAMPir_Composite_DB_No_Red_98%	25,264,871	3,009,412,726	11	119.1	24,292
AMPir_Composite_DB	2,102,697	141,704,428	45	67.4	627
AMPir_Composite_DB_No_Dup	2,072,171	139,648,678	45	67.4	627
AMPir_Composite_DB_No_Red_98%	1,939,076	130,783,896	45	67.4	627

Table S4. Statistics for each mining step (peptides, AMPs and non-haemolytic non-toxic AMPs). Min – Minimum. Av – Average. Max – Maximum. db – database; aspn - AspN, chym - Chymotrypsin, gluc - GluC, protk - Proteinase K and tryp – Trypsin.

Proteolysis Protocol	Peptides					AMPs					Non-Haemolytic and Non-Toxic AMPs				
	Number of sequences	Sum of lengths	Min. length	Av. length	Max. length	Number of sequences	Sum of lengths	Min. length	Av. length	Max. length	Number of sequences	Sum of lengths	Min. length	Av. length	Max. length
db1_aspn	4,376	81,416	11	18.6	40	214	4,188	11	19.6	38	9	184	12	20.4	33
db1_chym	2,696	40,761	11	15.1	40	35	639	11	18.3	36	3	64	16	21.3	31
db1_gluc	4,447	84,524	11	19	40	298	7,007	11	23.5	40	20	380	12	19	38
db1_protk	175	2,158	11	12.3	20	0	0	0	0	0	0	0	0	0	0
db1_tryp	4,318	78,289	11	18.1	40	49	857	11	17.5	34	4	54	12	13.5	17
db2_aspn	698	12,6	11	18.1	40	31	588	11	19	34	0	0	0	0	0
db2_chym	434	6,55	11	15.1	35	7	127	14	18.1	23	0	0	0	0	0
db2_gluc	744	14,292	11	19.2	40	47	1,115	11	23.7	38	1	18	18	18	18
db2_protk	25	318	11	12.7	22	1	14	14	14	14	0	0	0	0	0
db2_tryp	670	12,251	11	18.3	39	11	203	12	18.5	35	1	23	23	23	23
db3_aspn	1,820,209	33,705,927	11	18.5	40	134,007	2,591,695	11	19.3	40	5,055	102,719	11	20.3	40
db3_chym	818,622	11,676,951	11	14.3	40	14,534	247,057	11	17	40	946	16,993	11	18	40
db3_gluc	1,609,672	32,461,771	11	20.2	40	78,25	1,893,586	11	24.2	40	4,974	97,263	11	19.6	40
db3_protk	58,164	715,16	11	12.3	38	456	6,549	11	14.4	38	9	126	11	14	19
db3_tryp	1,849,069	33,133,654	11	17.9	40	38,029	651,794	11	17.1	40	4,023	65,665	11	16.3	40
db4_aspn	586,367	10,802,126	11	18.4	40	42,756	812,875	11	19	40	1,486	29,222	11	19.7	40
db4_chym	250,336	3,542,070	11	14.1	40	4,26	70,45	11	16.5	40	269	4,579	11	17	31
db4_gluc	519,668	10,417,513	11	20	40	24,7	585,475	11	23.7	40	1,586	29,954	11	18.9	40
db4_protk	17,08	208,892	11	12.2	37	111	1,539	11	13.9	22	3	46	12	15.3	20
db4_tryp	595,91	10,654,503	11	17.9	40	12,479	210,986	11	16.9	40	1,356	21,75	11	16	37
db5_aspn	996,612	18,503,524	11	18.6	40	72,434	1,394,948	11	19.3	40	2,633	53,244	11	20.2	40
db5_chym	436,127	6,201,383	11	14.2	40	3,929	66,532	11	16.9	40	234	4,082	11	17.4	38
db5_gluc	883,537	17,852,510	11	20.2	40	40,721	980,619	11	24.1	40	2,64	51,332	11	19.4	40
db5_protk	30,722	377,017	11	12.3	38	239	3,398	11	14.2	34	11	150	11	13.6	19
db5_tryp	1,014,421	18,203,744	11	17.9	40	20,694	352,647	11	17	40	2,134	34,286	11	16.1	40
db6_aspn	177,282	3,251,508	11	18.3	40	12,861	240,727	11	18.7	40	389	7,5	11	19.3	40
db6_chym	69,353	965,782	11	13.9	40	1,162	18,719	11	16.1	39	70	1,158	11	16.5	33
db6_gluc	153,954	3,102,010	11	20.1	40	6,591	153,405	11	23.3	40	416	7,489	11	18	38
db6_protk	4,704	56,995	11	12.1	23	21	297	11	14.1	23	0	0	0	0	0
db6_tryp	179,035	3,182,422	11	17.8	40	3,8	63,469	11	16.7	40	375	5,831	11	15.5	39
db7_aspn	97,943	1,804,776	11	18.4	40	6,914	135,987	11	19.7	40	299	6,292	11	21	39
db7_chym	49,536	717,004	11	14.5	40	937	16,47	11	17.6	38	54	1,064	11	19.7	35
db7_gluc	86,699	1,743,390	11	20.1	40	4,444	110,064	11	24.8	40	320	6,579	11	20.6	40
db7_protk	3,909	48,182	11	12.3	27	45	682	11	15.2	25	0	0	0	0	0
db7_tryp	100,524	1,809,687	11	18	40	2,029	35,385	11	17.4	40	208	3,577	11	17.2	34

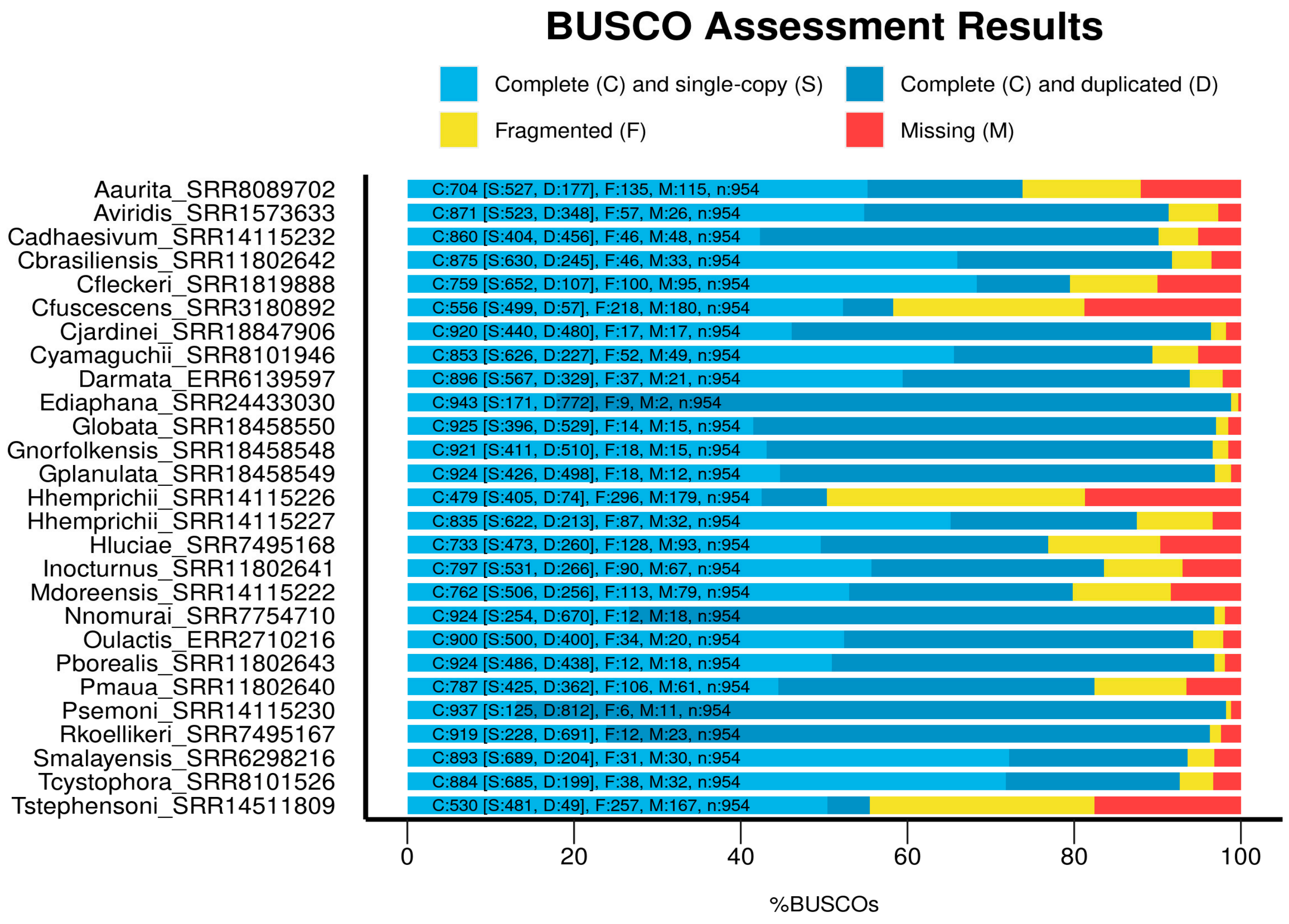

Figure S1. Completeness scores of the 27 assembled transcriptomes obtained from SRA data (NCBI), assessed with BUSCO using the Metazoa dataset. The chart shows the proportion of complete, fragmented, and missing gene copies for each transcriptome.

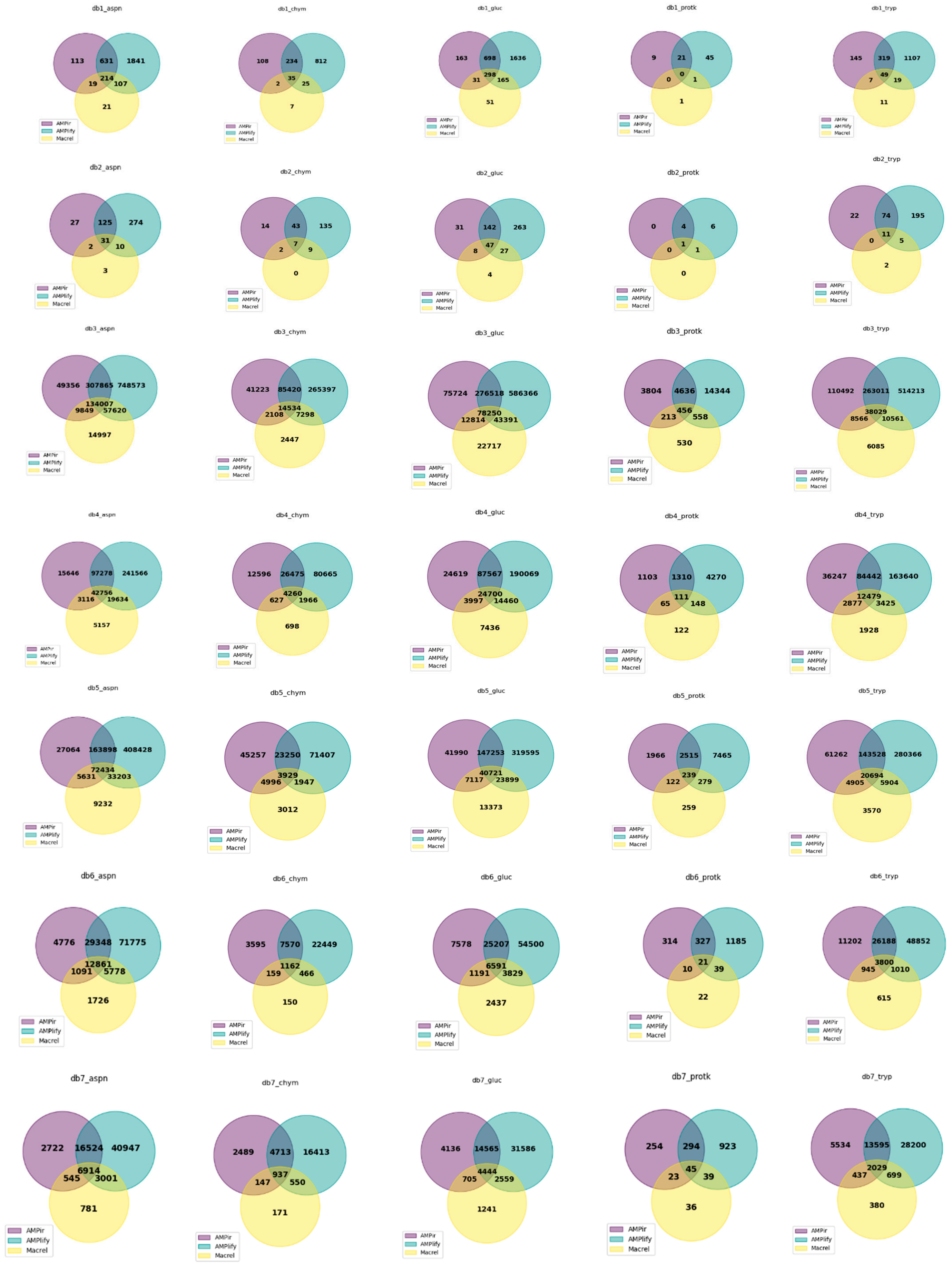

Figure S2. Venn Diagrams representing the number of predicted AMPs by AMPir (pink), AMPlify (blue) and Macrel (yellow) from each protease-database combination. db – database; aspn - AspN, chym - Chymotrypsin, gluc - GluC, protk - Proteinase K and tryp – Trypsin.

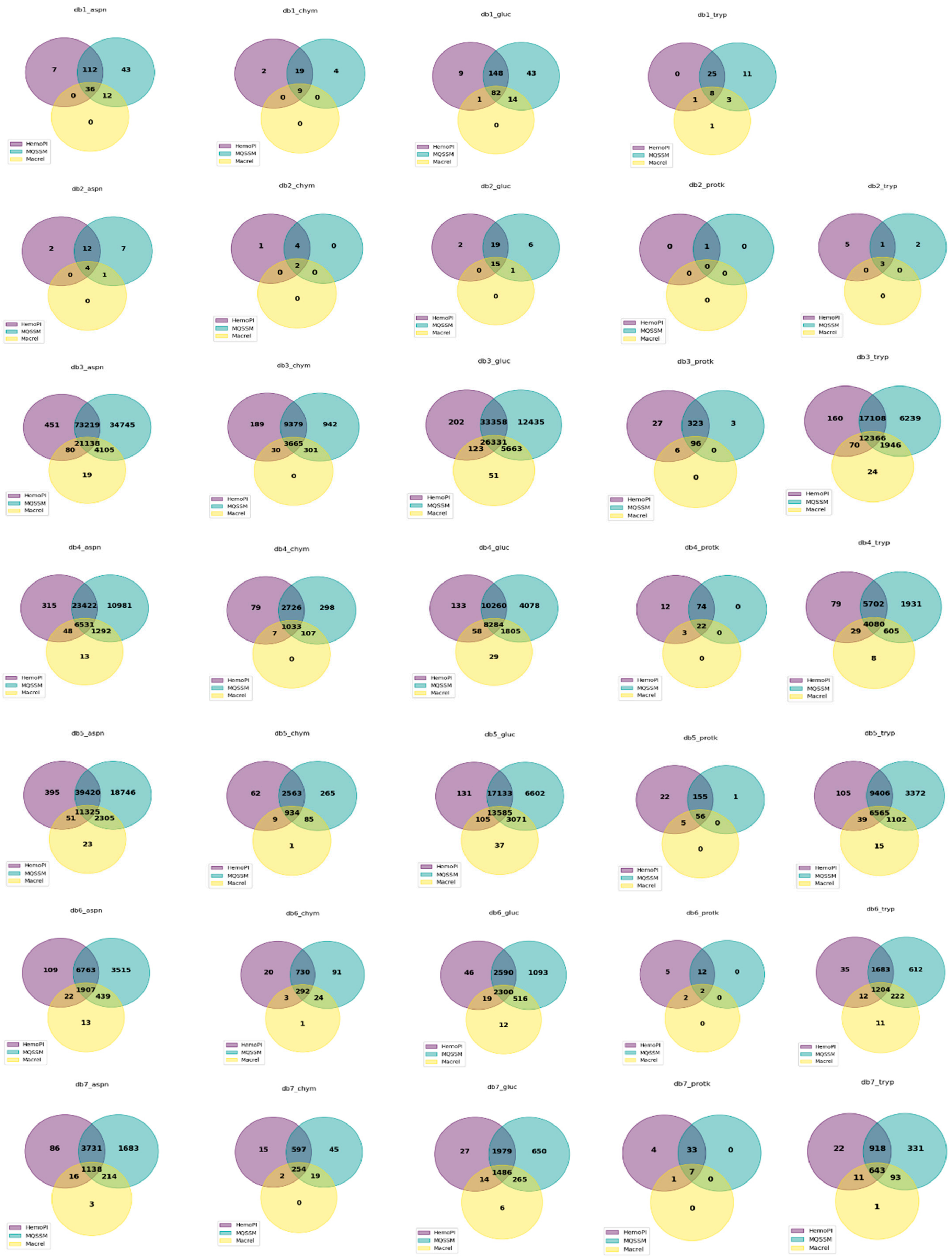

Figure S3. Venn Diagrams representing the number of predicted non-haemolytic AMPs by HemoPi (pink), MQSSM (blue) and Macrel (yellow) from each protease-database pair. db – database; aspn - AspN, chym - Chymotrypsin, gluc - GluC, protk - Proteinase K and tryp – Trypsin.

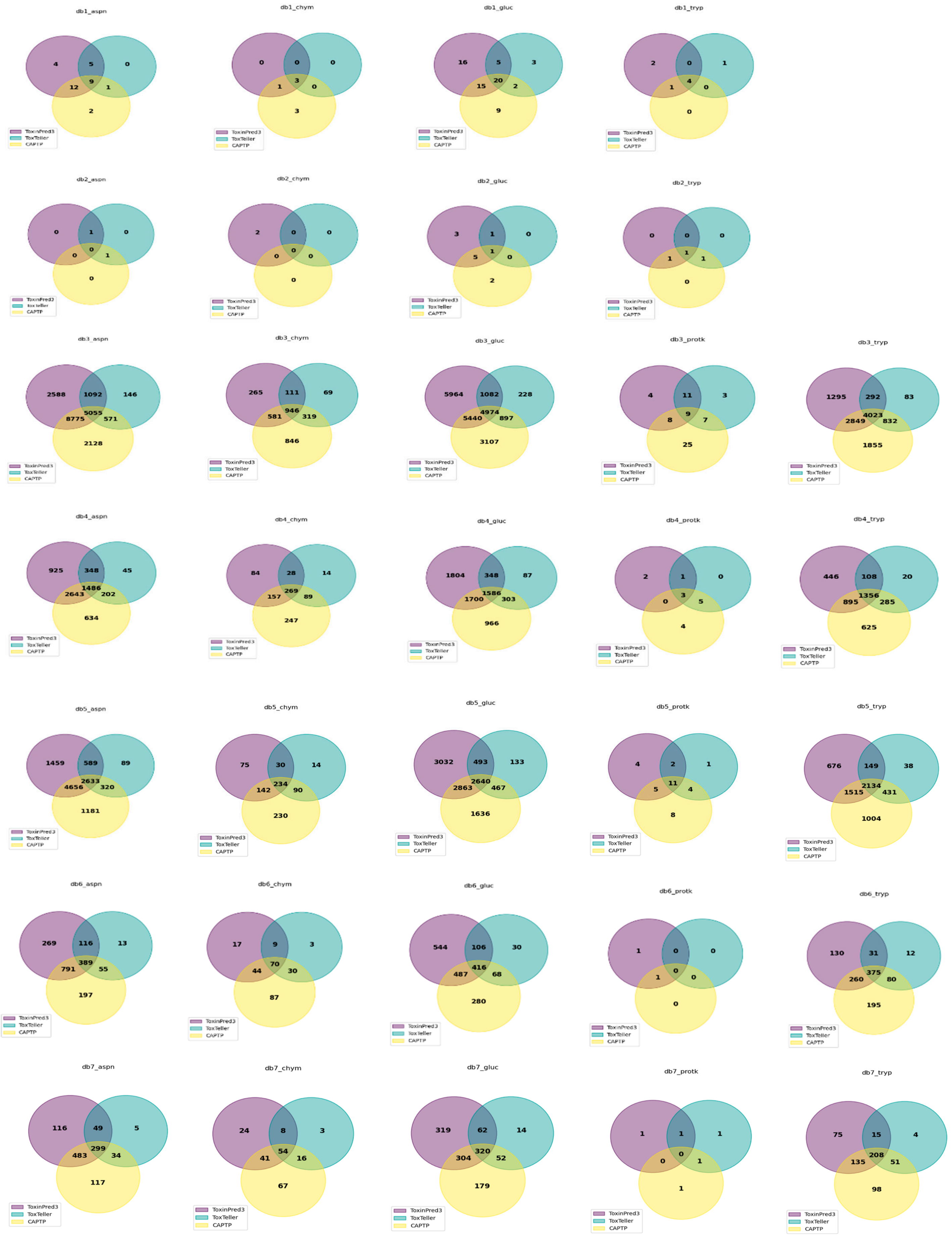

Figure S4. Venn Diagrams representing the number of predicted non-haemolytic and non-toxic AMPs by ToxinPred3 (pink), ToxTeller (blue) and CAPTP (yellow) from each protease-database pair. db – database; aspn - AspN, chym - Chymotrypsin, gluc - GluC, protk - Proteinase K and tryp – Trypsin.

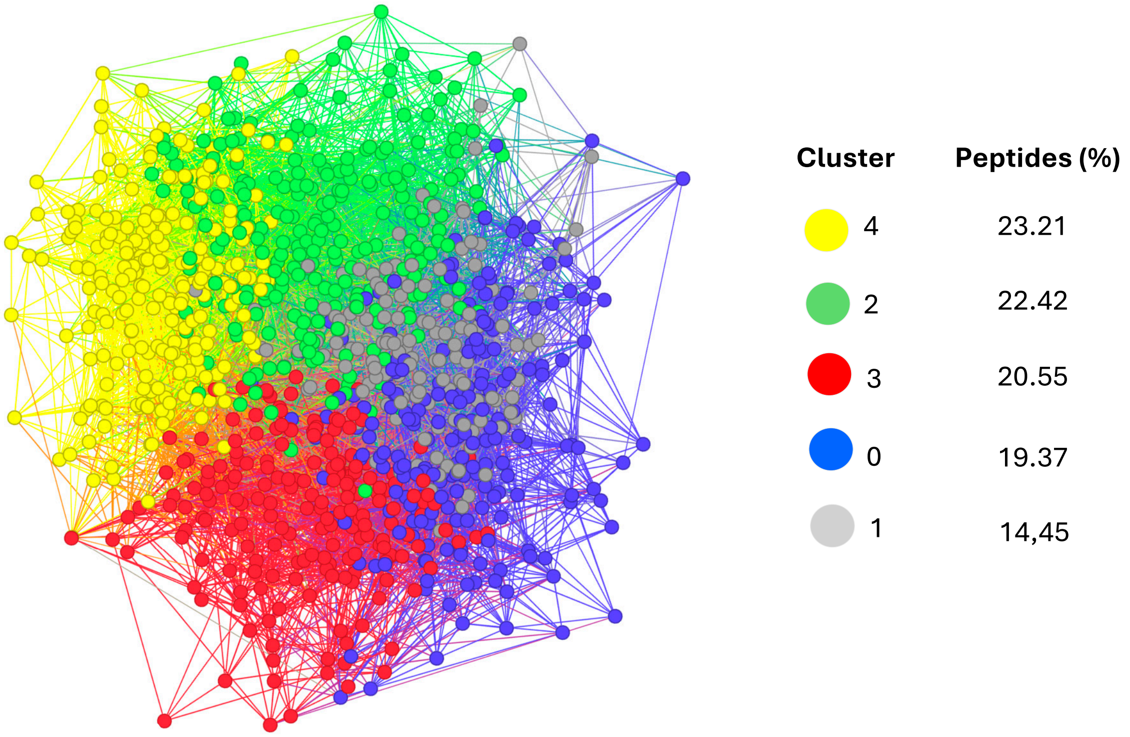

Figure S5. HSPN clustering of the 1,017 AMPs obtained from the intersection of the Harmonic and Hub-Bridge datasets within the Cnidaria Singular AMPs (CnSA). Distinct clusters are highlighted in different colours.

a

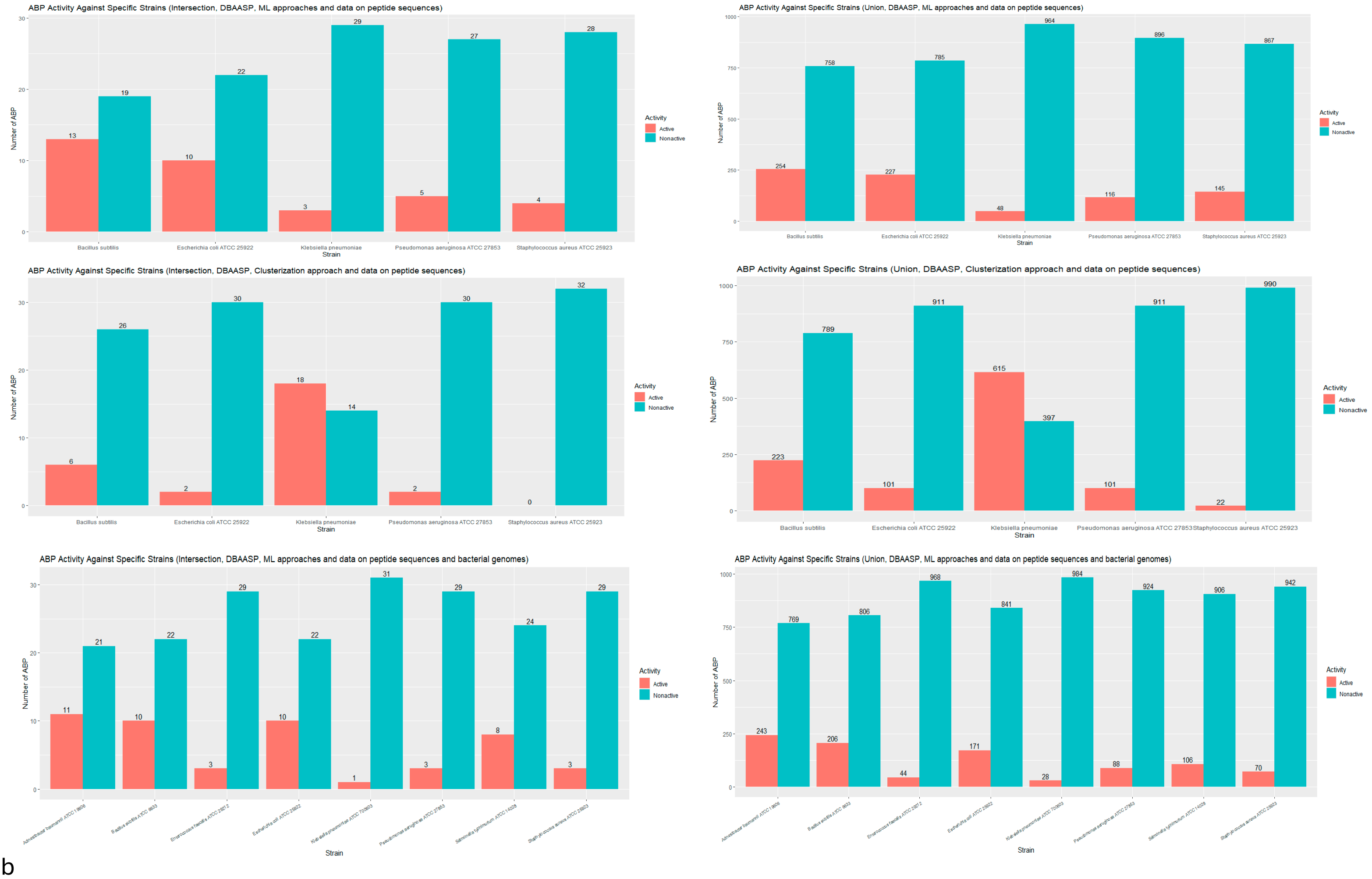

b

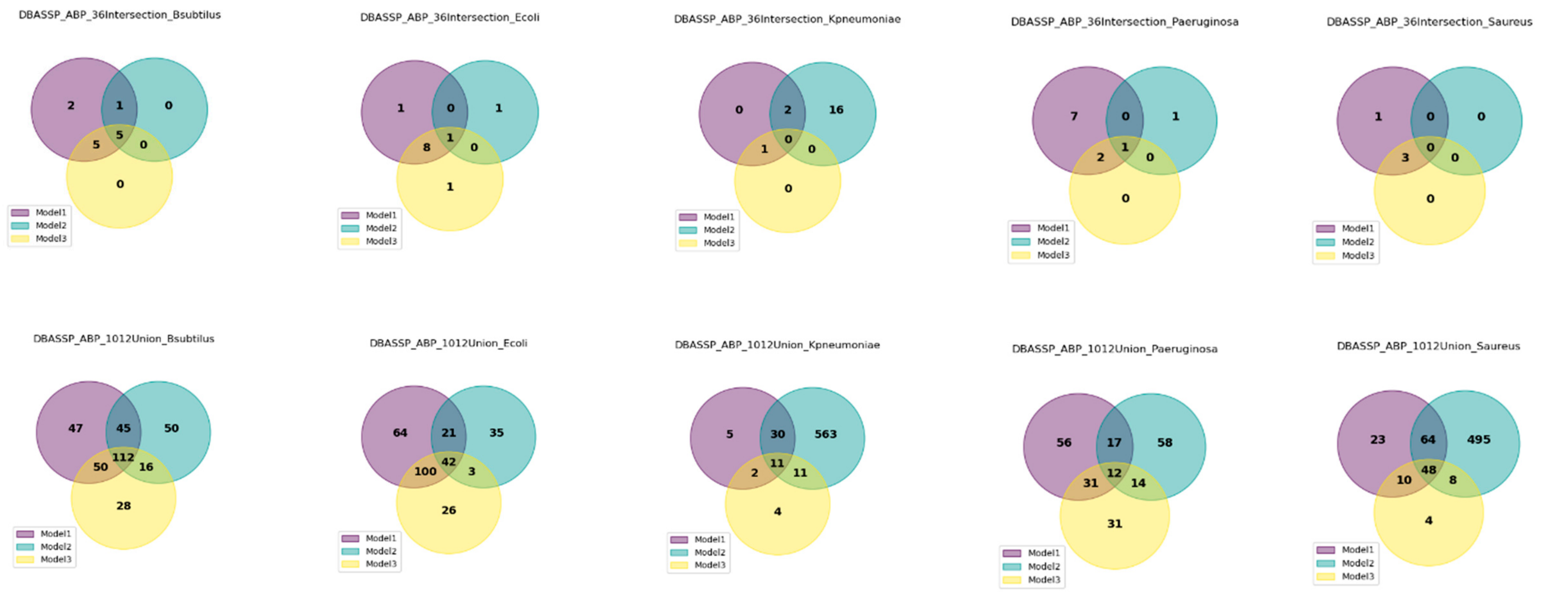

Figure S6. a) Strain-specific activity predictions for ABPs derived from the most representative CnSA datasets. Predictions were performed for both the union dataset (1,012 peptides) and the intersection dataset (32 peptides). Three models from DBAASP were used - Model I: ML predictions based on AMP sequence data; Model II: Cluster-based predictions using peptide sequence data; Model III: ML predictions integrating peptide and bacterial genome data. Results are represented in the boxplot, with red boxes indicating peptides predicted as active and blue boxes indicating non-active peptides for specific bacterial strains. **b)** Venn diagrams summarizing activity predictions for five bacterial strains - *Bacillus subtilis, Escherichia coli, Klebsiella pneumoniae, Pseudomonas aeruginosa* and *Staphylococcus aureus*. The diagrams illustrate the overlap of the strain-specific activity predictions from Figure 8a. These analyses were used to rank the potential activities of Cnidaria-derived ABPs against the selected strains.

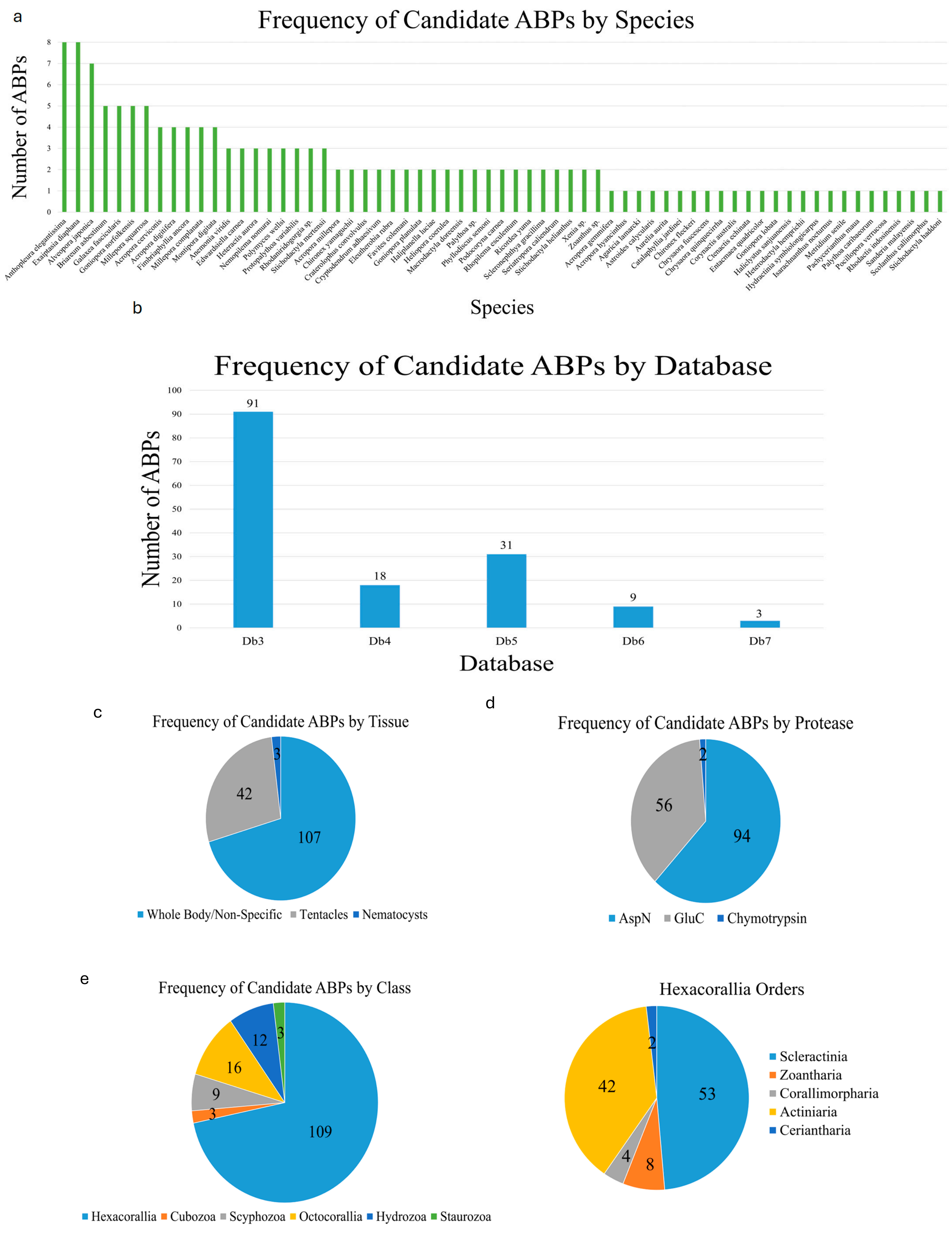

Figure S7. Distribution of the 152 predicted antimicrobial peptides (ABPs) across various categories: **a)** Cnidaria species; **b)** database origin; **c)** tissue type; **d)** protease associated with the in silico proteolysis; and **e)** Cnidarian classes, with a focus on Hexacorallia orders (Scleractinia, Zoantharia, Corallimorpharia, Actiniaria and Ceriantharia).

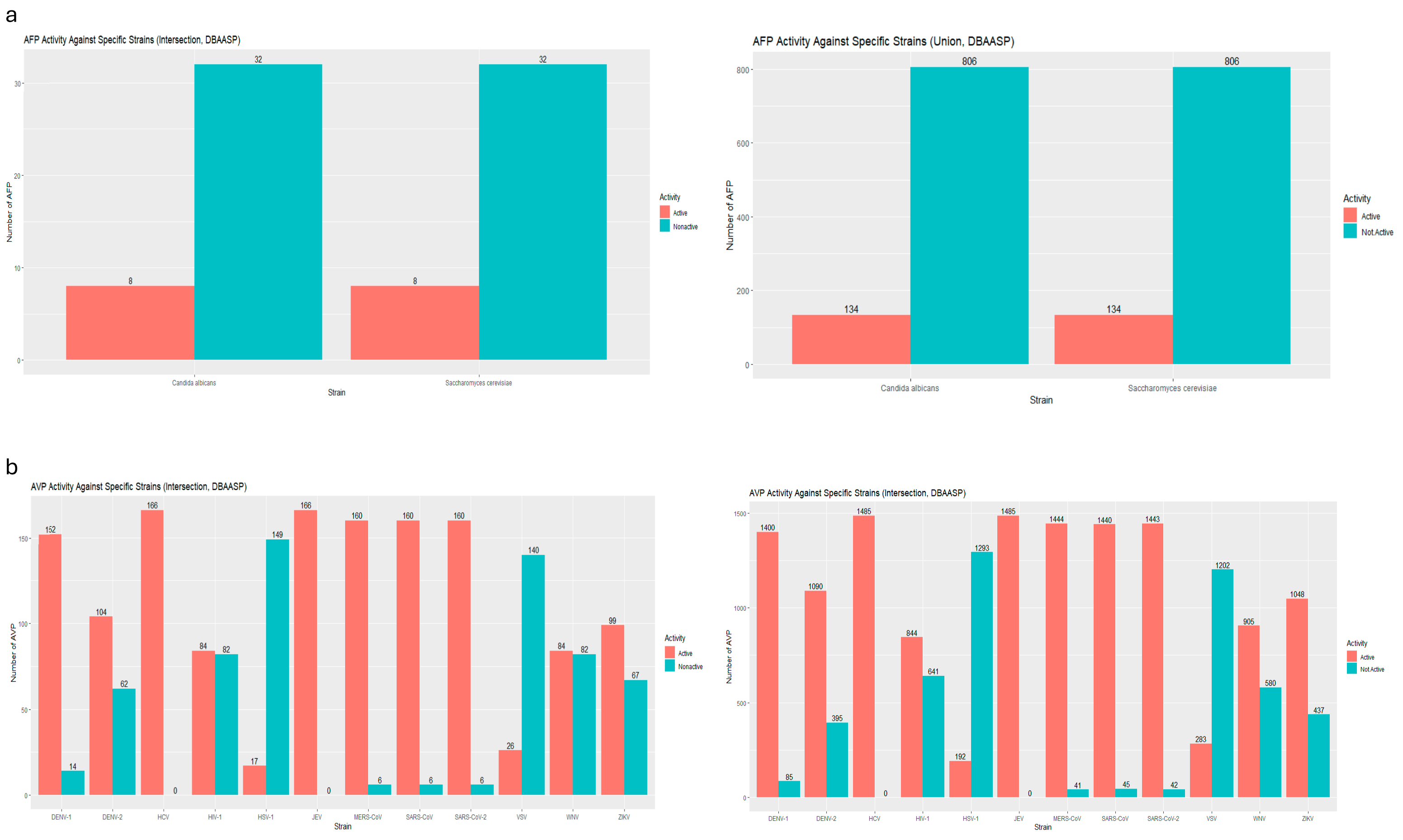

Figure S8. a) Antifungal specific predictions; and **b)** Antiviral specific predictions. Predictions were made for both intersection and union datasets in DBAASP.
